# Stress‐Dispersed Superstructure of Sn_3_(PO_4_)_2_@PC Derived from Programmable Assembly of Metal–Organic Framework as Long‐Life Potassium/Sodium‐Ion Batteries Anodes

**DOI:** 10.1002/advs.202206587

**Published:** 2023-04-23

**Authors:** Huimin Jiang, Shuo Zhang, Liting Yan, Yanlong Xing, Zhichao Zhang, Qiuju Zheng, Jianxing Shen, Xuebo Zhao, Lianzhou Wang

**Affiliations:** ^1^ School of Materials Science and Engineering Qilu University of Technology (Shandong Academy of Sciences) Jinan 250353 P. R. China; ^2^ State Key Laboratory of Heavy Oil Processing College of Chemistry and Chemical Engineering China University of Petroleum (East China) Qingdao 266580 P. R. China; ^3^ Key Laboratory of Emergency and Trauma Ministry of Education Hainan Medical University Haikou 571199 P. R. China; ^4^ Tianmu Lake Institute of Advanced Energy Storage Technologies Co., Ltd Liyang 213300 P. R. China; ^5^ School of Chemical Engineering and Australian Institute for Bioengineering and Nanotechnology University of Queensland St Lucia QLD 4072 Australia

**Keywords:** controllable particle attachment crystallization strategy, metal–organic framework, potassium‐ion battery, Sn_3_(PO_4_)_2_ superstructure, sodium‐ion battery

## Abstract

The structures of anode materials significantly affect their properties in rechargeable batteries. Material nanosizing and electrode integrity are both beneficial for performance enhancement of batteries, but it is challenging to guarantee optimized nanosizing particles and high structural integrity simultaneously. Herein, a programmable assembly strategy of metal–organic frameworks (MOFs) is used to construct a Sn‐based MOF superstructure precursor. After calcination under inert atmosphere, the as‐fabricated Sn_3_(PO_4_)_2_@phosphorus doped carbon (Sn_3_(PO_4_)_2_@PC‐48) well inherited the morphology of Sn‐MOF superstructure precursor. The resultant new material exhibits appreciable reversible capacity and low capacity degradation for K^+^ storage (144.0 mAh g^−1^ at 5 A g^−1^ with 90.1% capacity retained after 10000 cycles) and Na^+^ storage (202.5 mAh g^−1^ at 5 A g^−1^ with 96.0% capacity retained after 8000 cycles). Detailed characterizations, density functional theory calculations, and finite element analysis simulations reveal that the optimized electronic structure and the stress‐dispersed superstructure morphology of Sn_3_(PO_4_)_2_@PC promote the electronic conductivity, enhance K^+^/ Na^+^ binding ability and improve the structure stabilization efficiently. This strategy to optimize the structure of anode materials by controlling the MOF growth process offer new dimension to regulate the materials precisely in the energy field.

## Graphical abstract

A controllable particle attachment crystallization strategy of metal–organic frameworks (MOFs) is used to construct Sn_3_(PO_4_)_2_ superstructure as long‐life potassium/sodium‐ion batteries anodes. The storage mechanisms of Na^+^ and K^+^ in Sn_3_(PO_4_)_2_ are revealed for the first time. Furthermore, density functional theory (DFT) calculations and finite element analysis (FEA) simulations are conducted to analyze the underlying physicochemical mechanism.

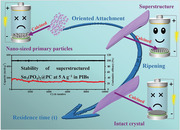

## 1 Introduction

The rapidly increasing demand for lithium‐ion batteries (LIBs) encounter great challenges, such as low abundance and heterogeneous distribution of lithium raw materials. Therefore, batteries based on other abundant alkaline metals, such as potassium‐ion batteries (PIBs) and sodium‐ion batteries(SIBs) have been considered a promising alternative to LIBs.^[^ ^1^ ^]^ At present, conversion‐alloying type anode materials exhibit high theoretical capacity for SIBs and PIBs have drawn great attention.^[^ ^1^, ^2^ ^]^ However, poor conductivity, severe volume expansion, and harmful pulverization of the materials during charge/discharge cycles seriously impact the cycle stability.

Prior studies have revealed that downsizing the feature size to the nanoscale allows the material to tolerance larger (de)sodium/ (de)potassium strains without fracture.^[^ ^3^ ^]^ Nanostructuring is beneficial to the life cycle of anode materials. However, it could introduce new fundamental challenges, including low Coulombic efficiency (CE), which could hinder ionic diffusion and electronic transportation due to the higher inter particle diffusion energy barrier. Moreover, electrical contact between the nanoparticles could be easily altered or diminished by the volume change during cycling while severely decreasing the cycle stability of the electrode. In different structures, the design of anode materials is essential to ensure both nanosizing particles and high structural integrity. The electrode materials must be composed of nanosized particles and they should be tightly integrated to ensure the stability of the overall structure. The superstructure materials integrated tightly by elementary nanograins exhibited great potential in meeting the structure requirements of anode materials.^[^ ^4^ ^]^ However, based on numerous investigations on the effect of superstructures as anode materials, no effective synthesis method has been developed for the controllable synthesis of superstructure nanomaterials.

Metal–organic frameworks (MOFs) and their derivatives have intrigued new interests for the construction of electrode materials with unrivaled tunability.^[^ ^5^ ^]^ The special morphologies of MOFs precursor are well kept after the thermal transformation and hence, the targeted materials after calcination can be fabricated by controlling the growth of MOFs precursor.^[^ ^6^ ^]^ As reported in our previously works, the crystallization by particle attachment (CPA) process for the growth of MOFs (HKUST‐1, MOF‐5, and NOTT‐100)^[^ ^7^ ^]^ can be applied to construct superstructures, which is mainly interpreted with three stages: 1) The generation of nanosized primary particles. The primary particles growth occurs at concentrations reach the concentration of nucleation. 2) The oriented attachment of primary particles into superstructures to reduce the surface energy and lowers the enthalpy of the system. 3) The Ostwald ripening of superstructures into single crystals. Inspired by the controllable CPA growing process of MOFs, we constructed a novel interconnected superstructure of Sn_3_(PO_4_)_2_@PC anodes derived from Sn‐based phosphate MOF superstructure precursor.

We found that excellent anode materials of nanosized superstructure could be achieved by controlling the CPA growth process of phosphonate‐based MOF precursors. At first, Sn‐based phosphate MOF superstructure precursor was synthesized by controlling the reaction stage of oriented tiny nanoarchitecture attachment. After calcination in an inert atmosphere, the unique morphology of the MOF superstructure precursor was well preserved. The resultant Sn_3_(PO_4_)_2_ exhibited a novel long‐range self‐assembled superstructure, which was built by the densest packing of numerous interconnected nanoparticles. In the superstructure, ultrasmall Sn_3_(PO_4_)_2_ nanocrystal (≈10 nm) was uniformly dispersed in the carbon matrix. An ideal anode material model having good conductivity and high stability was obtained. The obtained Sn_3_(PO_4_)_2_@PC demonstrated high capacity and long‐term cycle stability while acting as the anode for SIBs/PIBs. The reason was attributed to the positive effect of the unique hierarchical structure and excellent intrinsic performance of Sn_3_(PO_4_)_2_. The tight packing of interconnected nanosized primary building blocks in the superstructure guaranteed both small size and high structural integrity. This strategy balanced two conflicting roles, namely, keeping the unhindered ionic diffusion and electronic transportation, whilst reducing the volume expansion stress and harmful pulverization of the materials through decrease of the particle size. The storage mechanisms of Na^+^ and K^+^ in Sn_3_(PO_4_)_2_ were revealed through the combination of electrochemical kinetic analysis and a series of in situ and ex situ characterization tests, representing one of the first evidence‐based reports on the reaction mechanism of Sn_3_(PO_4_)_2_ used as the anode of SIBs and PIBs. Furthermore, density functional theory (DFT) calculations and finite element analysis (FEA) simulations were conducted to analyze the fabulous structural stability and the underlying mechanism of Sn_3_(PO_4_)_2_@PC superstructure. The excellent anode materials of nanosized superstructure was achieved in a creative way by controlling the CPA growth process of phosphonate‐based MOF precursors. This work offers a new and versatile route to produce superstructure anode materials with programmable functionalities and tailored geometry, which could be used in a broad range of applications.

## 2 Results and Discussion

**Figure 1:**
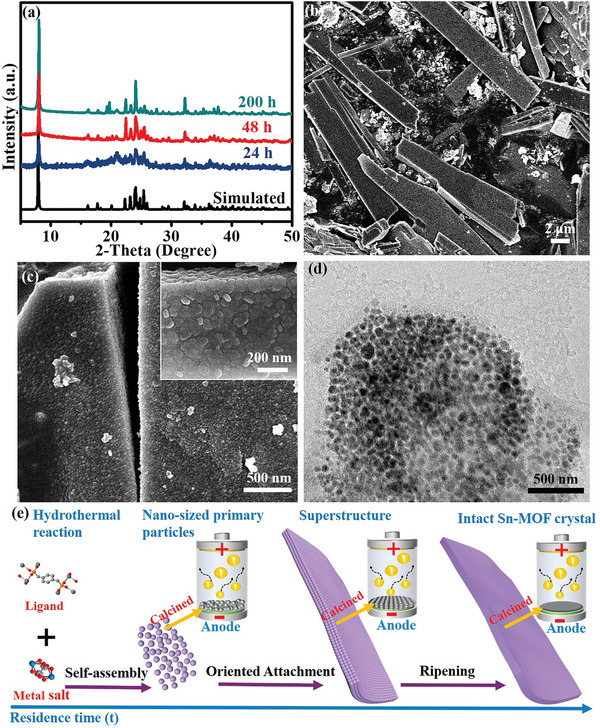
a) XRD patterns of Sn‐based MOFs hydrothermal for different time. b,c) SEM images of Sn‐MOF‐48. d) TEM image of Sn‐MOF‐48. e) Schematic illustration of the programmable assembly process for Sn‐MOF precursors and the applications as anode materials in batteries.

The Sn‐MOF prepared under different hydrothermal times was named Sn‐MOF‐T (24, 48, and 200 h) and was included in the Experimental Section of the Supporting Information. As shown in **Figure** **1**a, the X‐ray diffraction (XRD) profiles of Sn‐MOF‐24, Sn‐MOF‐48, and Sn‐MOF‐200 were consistent with the profile simulated from single‐crystal structure data, indicating the successful synthesis of the phosphorous‐containing Sn‐MOF precursors.^[^ ^8^ ^]^ Field emission scanning electron microscope (FESEM) image of Sn‐MOF‐24 showed a morphology of nanosized particles (Figure S1a,b, Supporting Information). Once the hydrothermal reaction time increased to 48 h, the obtained sample (Sn‐MOF‐48) demonstrated a higher degree of crystallinity. As shown in Figure 1b,c, the Sn‐MOF‐48 exhibited the apparent characteristics of a superstructure. Sn‐MOF‐48 consisted of regular‐shaped nanocrystals and the rugged primary nanocrystals were closely packed together while being stacked into a well‐defined superstructure. The obvious stripes and boundaries could be observed from the front and side views of the SEM images. The morphologies were also investigated by the transmission electron microscope (TEM). TEM image disclosed the presence of abundant nanosized particles in the Sn‐MOF‐48, which confirmed that the superstructure was built by the stacked of numerous nanosized particles (Figure 1d). With the increase in hydrothermal reaction time to 200 h, the small nanocrystals, as well as the characteristics of the superstructure disappeared. The flat surfaces of the solid bulk crystal were formed by the stacking and ripening of the primary nanocrystals (see magnified images in Figure S2, Supporting Information). In contrast to the morphologies of Sn‐MOF‐48, the Sn‐MOF‐200 exhibited a homogeneous structure having no obvious rugged grains. The boundary as observed in the TEM image (Figure S3, Supporting Information), indicated that the dislocations and defects present on the Sn‐MOF superstructure were gradually eliminated with the increase in hydrothermal time either through the rearrangement or recrystallization of primary particles under the effects of Ostwald ripening.^[^ ^6^, ^9^ ^]^ The detailed growth procedures of the hierarchical composite structure were schematically represented in Figure 1e. During the initial hydrothermal reaction period, the supersaturated solvent‐induced precipitation preferred fast nucleation, followed by the reduction in crystal growth with the rapid decline in solution concentration while resulting in the formation of numerous nanometer‐size primary particles. As the time was prolonged to 48 h, the further growth via aggregation resulted in the MOF of the superstructure and assembly of primary nanoparticles could be obtained. With the further increase in the reaction time up to 200 h, intact Sn‐MOF was generated by the Ostwald ripening of superstructures at the expense of metal ions, linkers and smaller nanoparticles. Sn‐MOF with different sizes and morphologies could be obtained by controlling the crystal growth duration. After calcination under inert atmosphere, the as‐fabricated Sn_3_(PO_4_)_2_@PC well inherited the morphologies of their respective Sn‐MOF precursors. Next we investigated the effect of three different morphologies on the performance of the anodes.

To investigate the surface area of Sn‐MOF at different stages of crystallization, N_2_ adsorptions at 77K were performed on the activated Sn‐MOF‐24, Sn‐MOF‐48, and Sn‐MOF‐200 samples. As shown in Figure S4a,b (Supporting Information), pore size distribution revealed the presence of mesopores centered at 2.0–30 nm in Sn‐MOFs. These mesopores could have originated from the void space present between the orderly packed primary nanoparticles in the superstructures.^[^ ^7^ ^]^ Elongating the residence time, the mesopore volumes of resulting Sn‐MOFs gradually decreased. The variation in pore structure suggested that the mesoscale voids were filled by the crystals along with the ripening of superstructures. It was in satisfactory agreement with the SEM results (Figure 1b,c; Figure S2a,b, Supporting Information).

**Figure 2:**
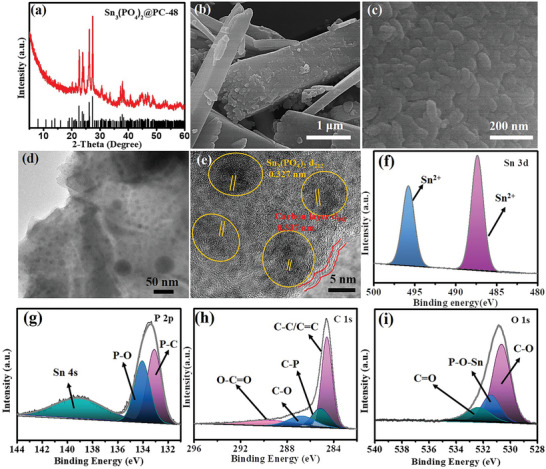
a) XRD pattern of Sn_3_(PO_4_)_2_@PC‐48. b,c) FESEM images of Sn_3_(PO_4_)_2_@PC‐48. d,e) TEM and HRTEM images of Sn_3_(PO_4_)_2_@PC‐48. f–i) High resolution XPS of Sn 3*d*, P 2*p*, C1*s*, and O 1*s* of Sn_3_(PO_4_)_2_@PC‐48.

The Sn_3_(PO_4_)_2_ phase was formed after annealing at 630 °C of Sn‐MOF precursor under the argon atmosphere as shown in **Figure** **2**a; Figures S5 and S6 (Supporting Information). The standard XRD pattern was shown as a reference. All the diffraction peaks could coincide with the Sn_3_(PO_4_)_2_ phase (JCPDS No. 70‐0391) with no impurity phases. Sn_3_(PO_4_)_2_@PC‐24, Sn_3_(PO_4_)_2_@PC‐48, and Sn_3_(PO_4_)_2_@PC‐200 were obtained through the calcination of as‐prepared Sn‐MOF‐24, Sn‐MOF‐48, and Sn‐MOF‐200 precursor under argon atmosphere, respectively. FESEM images of Sn_3_(PO_4_)_2_@PC‐24 (Figure S7a, Supporting Information), Sn_3_(PO_4_)_2_@PC‐48 (Figure 2b,c), and Sn_3_(PO_4_)_2_@PC‐200 (Figure S7b, Supporting Information) demonstrated that both samples possessed the regular morphologies similar to that of the corresponding Sn‐MOF precursor, indicating that the initial morphologies of precursors were well maintained after pyrolysis. The detailed structural information of Sn_3_(PO_4_)_2_@PC‐48 was further investigated by TEM and high‐resolution TEM (HRTEM). As shown in Figure 2d, the TEM image of Sn_3_(PO_4_)_2_@PC‐48 demonstrated the formation of a novel superstructure skeleton by the close aggregation of numerous interlinked primary nanoparticles. The ultrasmall Sn_3_(PO_4_)_2_ nanocrystals (≈10 nm) in the Sn_3_(PO_4_)_2_@PC‐48 were uniformly dispersed in the carbon matrix (Figure 2e). The mechanically robust conductive carbon layer was uniform throughout the superstructure while acting as a buffer to accommodate acute interior stress during the fracture of nanoparticles. There were clear crystal planes with a *d*‐spacing of 0.327 nm, which was consistent with the (212) plane of Sn_3_(PO_4_)_2_. The Sn_3_(PO_4_)_2_ was synthesized successfully with a high degree of crystallinity. The lattice fringes with the spacing of 0.337 nm were assigned to the (002) carbon plane. A uniform distribution without any remarkable aggregations of P, O, C, and Sn elements was observed through TEM‐energy dispersive X‐ray (EDX) spectroscopy (Figure S8, Supporting Information). The TEM image of Sn_3_(PO_4_)_2_@PC‐24 (Figure S9a, Supporting Information) revealed that tiny Sn_3_(PO_4_)_2_ nanocrystals (≈10 nm) were uniformly dispersed in the carbon matrix of Sn_3_(PO_4_)_2_@PC‐24. Whereas, a lack of macroscopic linking between the nanoparticle was witnessed. In contrast, the TEM image confirmed that the Sn_3_(PO_4_)_2_ showed obvious aggregation in Sn_3_(PO_4_)_2_@PC‐200 and non‐uniform Sn_3_(PO_4_)_2_ was irregularly dispersed in the carbon matrix (Figure S9b, Supporting Information).

To accurately determine the valence state and composition of the Sn_3_(PO_4_)_2_@PC‐48 samples, X‐ray photoelectron spectroscopy (XPS) measurements were performed. As shown in Figure S10 (Supporting Information), the XPS survey scan spectrum demonstrated the presence of Sn, P, C, and O elements in Sn_3_(PO_4_)_2_@PC‐48. The high‐resolution spectra of these elements confirmed the formation of Sn_3_(PO_4_)_2_@PC. There was a pair of peaks with an energy of 496.2 and 487.5 eV, which can be assigned to Sn 3*d* 3/2 and Sn 3*d* 5/2 in the Sn 3*d* spectra (Figure 2f), which implying the existence of the Sn element in the 2^+^ states.^[^ ^10^ ^]^ As illustrated in Figure 2g, the peak at 133.2 eV belonged to the P—C, the peak at 134.0 eV was assigned to P 2*p* from P—O and hence, the P species existed as PO_4_^3−^ in Sn_3_(PO_4_)_2_@PC.^[^ ^11^ ^]^ The peak at 139.0 eV could be assigned to the interfere of Sn 4*s* due to the very close binding energy between P 2*p* and Sn 4*s*. A similar phenomenon has also been reported previously in the literature.^[^ ^11^ ^]^ As presented in Figure 2h, the C 1*s* spectrum could be fitted into four peaks as O—C=O, C─O, C─P, and C—C/C=C groups at 289.2, 286.7, 285.1 and 284.6 eV, respectively. The presence of C—O, C─P, and O—C=O offered numerous anchoring sites for electrochemically active materials which inhibited particle agglomeration or disengagement from electrodes. The O 1*s* spectrum (Figure 2i) was deconvoluted into three signals while being ascribed to the C=O (530.9 eV), P—O—Sn (531.5 eV) of Sn_3_(PO_4_)_2_, and C—O (532.3 eV).^[^ ^11^, ^12^ ^]^ Raman spectra in Figure S11 (Supporting Information) presented *D* (≈1350 cm^−1^) and G (≈1591 cm^−1^) bands with the intensity ratios (*I*_D_/*I*_G_) of 1.33, which confirmed the existence of graphitized and amorphous carbon in Sn_3_(PO_4_)_2_@PC‐48 composite. The *D*‐band with a wide bandwidth suggested that sufficient defects were present to facilitate K and Na ion transport to Sn_3_(PO_4_)_2_. The high surface area is favorable to improve the performance by not only increases the surface interaction, but also provides an additional long‐range ion transport channel. The N_2_ sorption isotherms (Figure S12, Supporting Information) suggested that Sn_3_(PO_4_)_2_@PC‐48 possessed a moderate specific surface area of 7.12 m^2^ g^−1^. To further understand the transformation and formation process of Sn_3_(PO_4_)_2_@PC during calcination, the thermogravimetric analysis (TGA) of Sn‐MOF‐48 was conducted separately in air and nitrogen atmosphere (Figure S13, Supporting Information). The TGA curve of Sn‐MOF‐48 operated under nitrogen atmosphere present three stages. During the temperature range of 30–380°C, the quality reduced by ≈2.2%, which was attributed to the water and solvent desorption. The second temperature stage ranging from 490 to 570°C, which was due to the endothermic process accompanied by the formation of Sn_3_(PO_4_)_2_. In the third stage (>690°C), the resulting intermediate was further converted into Sn, which was demonstrated by the XRD outcome (Figure S14, Supporting Information). As shown in Figure S14 (Supporting Information), the Sn‐MOF‐48 was heated to 850 and 900°C in an inert atmosphere. The phase of Sn‐MOF‐48 was converted into Sn (JCPDS No. 04‐0673) after calcined for 2 h under inert atmosphere. During the first decomposition steps, almost no difference was observed in Sn‐MOF‐48 powder under air or inert atmosphere. In the second stage (470–600°C), the mass reduction was attributed to the conversion of Sn_3_(PO_4_)_2_ to SnO*_x_* in air. The TGA results indicated that Sn‐MOF was fully decomposed to Sn_3_(PO_4_)_2_ at a certain temperature range (570–690°C) under an inert atmosphere, which was consistent with the XRD results.

**Figure 3:**
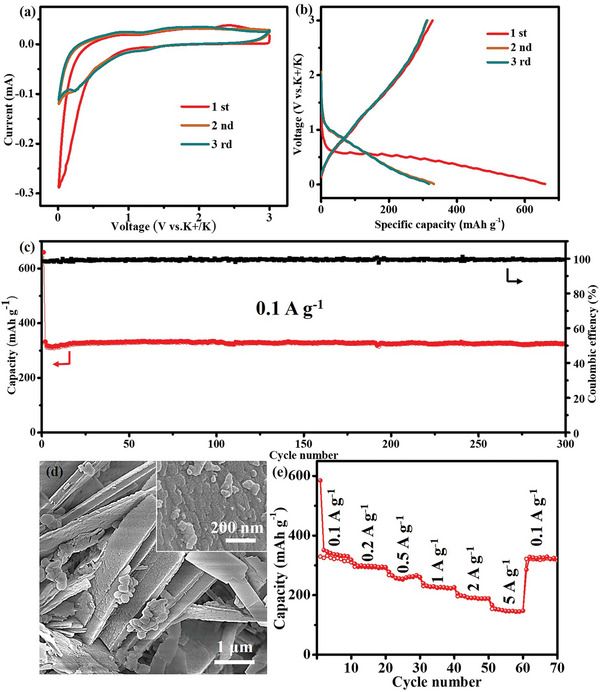
Electrochemical performances for PIBs. a) CV curves of the Sn_3_(PO_4_)_2_@PC‐48 electrode at a scan rate of 0.1 mV s^−1^ for the first three cycles. b) Galvanostatic discharge and charge profiles for the first three cycles of the Sn_3_(PO_4_)_2_@PC‐48 electrode at a current density of 0.1 A g^−1^. c) Cycle performances of the Sn_3_(PO_4_)_2_@PC‐48 electrodes at 0.1 A g^−1^. d) SEM images of Sn_3_(PO_4_)_2_@PC‐48 after 300 cycles. e) Rate performance of Sn_3_(PO_4_)_2_@PC‐48.

To understand the potassiation/depotassiation process, the electrochemical properties of the Sn_3_(PO_4_)_2_@PC‐48 in PIBs were studied by cyclic voltammetry (CV) and galvanostatic charge/discharge measurements. **Figure** **3**a showed the first three CV curves of the Sn_3_(PO_4_)_2_@PC‐48 at a scan rate of 0.1 mV s^−1^ between 0.01 and 3.0 V. During the first cathodic process, a tiny bump appeared between 0.5 to 0.8 V, which might be attributed to the conversion reaction of Sn_3_(PO_4_)_2_, the alloying reaction of Sn and the growth of solid electrolyte interphase (SEI). Subsequently, the formed Sn alloys with K produced a cathodic peak at ≈0.25 V. The presence of another cathodic peak in the potential range of 0.10–0.20 V could be attributed to further alloying reaction and the intercalation of K^+^ into carbon. During the anodic scan, a wide anodic peak from 2.7 to 1.2 V was observed which could be due to the depotassiation of K—Sn and K—PO_4_. The CV curves overlap very well starting from the second cycle, which suggested the formation of steady SEI film and high reversibility of the Sn_3_(PO_4_)_2_@PC‐48 electrode.

The galvanostatic charge/ discharge profiles of the Sn_3_(PO_4_)_2_@PC‐48 electrode at a current density of 0.1 A g^−1^ was presented in Figure 3b. The initial discharge and charge capacities of the Sn_3_(PO_4_)_2_@PC‐48 electrode were determined to be 659.8 and 328.6 mAh g^−1^, respectively. Meanwhile, the initial CE of the Sn_3_(PO_4_)_2_@PC‐48 electrode was found to be 49.8%. The low initial CE was due to the irreversible electrochemical reactions upon cycling, such as the formation of an SEI film, the reduction reaction of Sn_3_(PO_4_)_2_ and the trapping of K^+^ in the carbon matrix. Figure 3c demonstrated the cycle stability and CE at 0.1 A g^−1^ over 300 cycles. The initial capacity loss can be attributed to the inevitable formation of solid‐electrolyte interface (SEI) layer and initial irreversible potassium consumption. Sn_3_(PO_4_)_2_‐48 exhibited a reversible capacity of 324 mAh g^−1^ after 300 cycles at 0.1 A g^−1^ while providing capacity retention close to 100% (99.7%). Simultaneously, the CE rapidly increased up to 98.6% during the second cycle (Figure 3c). In comparison, most of the anode materials undergo hundreds of cycles for the CE to reach >99%. The average CE of the Sn_3_(PO_4_)_2_‐48 for the 1st to 300th cycles was as high as 99.6%. At a relatively slow rate (0.1 C), the CE was found to be superior compared to previous reports. High CEs are critical for the operation of a battery. We further tested the performance of Sn_3_(PO_4_)_2_@PC‐48 with active material mass loading up to 3.17 mg cm^−2^. At a current density of 100 mA g^−1^, the Sn_3_(PO_4_)_2_@PC‐48 electrode exhibited reversible capacity of 309 mAh g^−1^. The capacity retention was as high as 95.4% after 300 cycles The specific capacity of the high mass loading cell (309 mAh g^−1^) was only slightly lower than that of the low mass loading cell shown in Figure 3c (324 mAh g^−1^), which indicated that almost all the Sn_3_(PO_4_)_2_@PC‐48 is active in the thick electrode.

The good reversibility indicated that the unique superstructure of Sn_3_(PO_4_)_2_@PC‐48 benefited the stability of the electrode during cycling, which was confirmed via SEM and TEM characterization after 300 cycles (Figure 3d; Figure S16, Supporting Information). As shown in Figure 3d, the superstructure morphology was preserved after cycling. The ultrasmall Sn_3_(PO_4_)_2_ nanocrystals (≈10 nm) in the Sn_3_(PO_4_)_2_@PC‐48 were uniformly dispersed in the carbon matrix (Figure S16, Supporting Information). The robust carbon layer was uniform throughout the superstructure. The nanosized particle could effectively resolve the critical issues of severe volume expansion and prevent fracture. The mechanically robust carbon framework remained uniform throughout the curved regions, which acted as a buffer to accommodate acute interior stresses during potassium.^[^ ^13^ ^]^ The interlinked carbon framework worked as an electrical highway and a mechanical backbone so that all nanoparticles could be electrochemically active. In order to further elucidate the structural and electronic properties of *P*‐doped carbon during the charging and discharging, we added the in situ Raman in Figure S17 (Supporting Information) to test the K^+^ storage in the *P*‐doped carbon through the first charge–discharge cycle. Intercalation of K guest into carbonaceous host strongly affects the position, shape and intensity of the *G*‐band at ≈1582 cm^−1^.^[^ ^14^ ^]^ However, there was no obvious difference of Raman spectra during the charge–discharge cycle (Figure S17, Supporting Information). The results indicated that the capacity of Sn_3_(PO_4_)_2_@PC‐48 was mainly produced by the Sn_3_(PO_4_)_2_@PC, the structure and defects of carbon network remained stable benefit from the well‐designed superstructure of Sn_3_(PO_4_)_2_@PC‐48.

The rate capabilities of the Sn_3_(PO_4_)_2_@PC‐48 electrodes were investigated by cycling the electrodes at different current densities in sequence (Figure 3e). The Sn_3_(PO_4_)_2_@PC‐48 electrode exhibited outstanding reversible capacities of 325, 295, 267, 231, 196, and 154 mAh g^−1^ at 0.1, 0.2, 0.5, 1, 2, and 5 A g^−1^, respectively. When the current density was reset to 0.1 A g^−1^, the capacity could recover.

**Figure 4:**
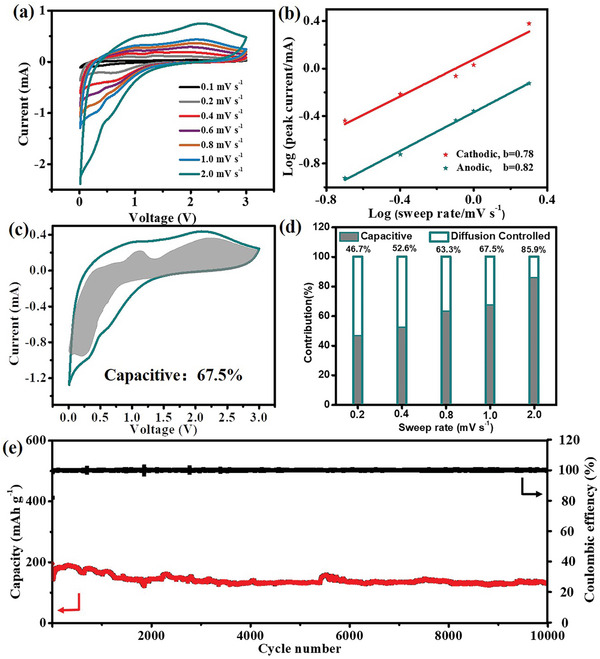
Electrochemical performances for PIBs. a) CV curves of the Sn_3_(PO_4_)_2_@PC‐48 electrode at various scan rate from 0.1 to 2 mV s^−1^. b) Plots of log (scan rate) versus log (peak current). c) Capacitive contribution in CV curves (shaded region) of Sn_3_(PO_4_)_2_@PC‐48. d) Contribution ratio of capacitive capacity in Sn_3_(PO_4_)_2_@PC‐48 at different sweep rates. e) Cycle performances of the Sn_3_(PO_4_)_2_@PC‐48 electrodes at 5 A g^−1^.

To further investigate the kinetics of potassium insertion/extraction into/from the Sn_3_(PO_4_)_2_@PC‐48 electrodes, a series of CV curves having different scan rates (0.1, 0.2, 0.4, 0.6, 0.8, 1.0, and 2.0 mV s^−1^) were recorded. As shown in **Figure** **4**a, a positive shift in the anodic peaks was observed with the increase in scan rates. The power‐law relationship between the measured peak current (*I*_p_) and the sweep rate (*ν*) could be calculated as follow:

(1) $$I_{p}=a\nu^{b}$$

where both *a* and *b* are adjustable parameters. The *b* reflects the control process of the electrochemical behavior, which could be determined from the slope of the plots (Figure 4b). The value of *b* approached 0.5 for a semi‐infinite diffusion‐controlled process, while it was close to 1.0 for a surface capacitive–controlled process. According to the outcomes, the values of *b* for the cathodic and anodic peaks were 0.82 and 0.78, respectively, which suggested the mixed contribution of the two processes in the Sn_3_(PO_4_)_2_@PC‐48 electrode. According to Dunn et al., the ratio of the two contributions could be estimated based on Equation 2.

(2) $$i=k_{1}v+k_{2}\nu^{1/2}$$

where *i* is the current at a fixed potential. The *k*_1_ *v* and *k*_2_ *v* ^1/2^ are the contributions of a surface‐controlled process and a diffusion‐controlled process, respectively. To simplify the calculation, the equation was reorganized as Equation 3 by dividing both sides by *v* ^1/2^.

(3) $$i/v^{1/2}=k_{1}v^{1/2}+k_{2}$$

In this equation, *k*_1_ and *k*_2_ could be easily obtained as the slope and intercept point at the *y*‐axis in a plot of *i/v* ^1/2^ versus *v* ^1/2^. Thus, the ratio of a diffusion‐controlled process and a surface‐controlled process at this potential could be clarified. As shown in Figure 4c, the capacitive process contributed 67.5% of the total capacity for a scan rate of 1.0 mV s^−1^. The contribution gradually decreased to 46.7% with the decline in scan rate up to 0.2 mV s^−1^ and increased up to 85.9% for the scan rate of 2.0 mV s^−1^ (Figure 4d).

To better understand the intrinsic performance of Sn_3_(PO_4_)_2_@PC‐48, its long‐term cycling performance was further investigated under low rate capacity (0.5 A g^−1^). As shown in Figure S18 (Supporting Information), Sn_3_(PO_4_)_2_@PC‐48 retained a reversible capacity of 260.3 mAh g^−1^ after 500 cycles with capacity retention of 99.4% and CE of 100%. The high CE and good electrochemical reversibility benefitted not only from the excellent intrinsic activity of Sn_3_(PO_4_)_2_, but also from the well‐designed carbon confinement nanocrystal superstructure of Sn_3_(PO_4_)_2_@PC‐48 electrode. Long‐term cycling stability of Sn_3_(PO_4_)_2_@PC‐24, Sn_3_(PO_4_)_2_@PC‐48, and Sn_3_(PO_4_)_2_@PC‐200 were further evaluated at 5 A g^−1^ (Figure 4e; Figures S19 and S20, Supporting Information). Even after 10000 cycles (Figure 4e), Sn_3_(PO_4_)_2_@PC‐48 displayed a capacity of 129.7 mAh g^−1^ at 5 A g^−1^ with a capacity retention of 90.1%, this performance is among the best reported so far (Table S2, Supporting Information). Whereas, Sn_3_(PO_4_)_2_@PC‐24 exhibited 34.2% retention after 5000 cycles (Figure S19, Supporting Information). As shown in Figure S20 (Supporting Information), the capacity of Sn_3_(PO_4_)_2_@PC‐200 decreased significantly with the retention of 65.2% after 5000 cycle. As per the outcomes, the excellent stability of Sn_3_(PO_4_)_2_@PC‐48 was resulted because of reasons, such as 1) the ultra‐small primary particles reduced the fracture risks effectively, 2) the interlinked mechanically robust framework of Sn_3_(PO_4_)_2_@PC‐48 superstructure worked as electrical highway while boosting ion storage, electron transportation kinetics and structural stability. The structure and defects remained stable during the repeat cycles. Therefore, compared to the Sn_3_(PO_4_)_2_@PC‐24 and Sn_3_(PO_4_)_2_@PC‐200, the Sn_3_(PO_4_)_2_@PC‐48 superstructure exhibited superior ion storage performance due to its highly integrated structure and small primary particles. To gain insights into the diffusion and reaction kinetics, electrochemical impedance spectroscopy (EIS) of the Sn_3_(PO_4_)_2_@PC‐48 electrode before and after 50 cycles at an open‐circuit voltage (Figure S21, Supporting Information) was measured and fitted by an equivalent circuit (the inset of Figure S21b, Supporting Information). The *R*_s_ indicated the resistance of the electrolyte and cell components. The *R*_f_ was the resistance related to the surface film, *R*_ct_ referred to the charge transfer resistance at the interfaces and *Z*_w_ represented the Warburg impedance related to K^+^ diffusion.^[^ ^15^ ^]^ The surface film resistance (*R*_f_), charge‐transfer resistance (*R*_ct_) and the Warburg impedance significantly decreased after successive cycles (Table S3, Supporting Information). It is noted that both *R*_f_ and *R*_ct_ of Sn_3_(PO_4_)_2_@PC‐48 electrode are much smaller after 50 cycles, implying the enhanced electrical conductivity, faster charge‐transfer kinetics and higher K^+^ diffusion ability of Sn_3_(PO_4_)_2_@PC‐48 electrode after the cycling. Without the unique morphology of superstructure, bulk Sn_3_(PO_4_)_2_@PC‐200 quickly fractured into unstable irregular pieces (Figure S22, Supporting Information) while resulting in severe capacity decay. The Sn_3_(PO_4_)_2_@PC‐24 had difficulty in ionic diffusion and electronic transportation due to the absence of macroscopic linking between the nanoparticle and higher interparticle diffusion energy barrier. Moreover, electrical contact between the nanoparticles was either easily altered or diminished by volume change during cycling while severely decreasing the cycle life of Sn_3_(PO_4_)_2_@PC‐24.

To further understand the electrochemical performance of Sn_3_(PO_4_)_2_@PC, the galvanostatic intermittent titration technique (GITT) was used to investigate the different charge/discharge levels. As shown in Figure S23 (Supporting Information), the open‐circuit potential (OCP) of Sn_3_(PO_4_)_2_@PC‐48 slowly, but steady decreased with the state of discharge. The opposite change occurred to the depotassiumsion process, where the OCP gradually increased as the charging proceeds. Compared to Sn_3_(PO_4_)_2_@PC‐24 and Sn_3_(PO_4_)_2_@PC‐200, the Sn_3_(PO_4_)_2_@PC‐48 exhibited a higher potassium ion diffusion coefficient.

**Figure 5:**
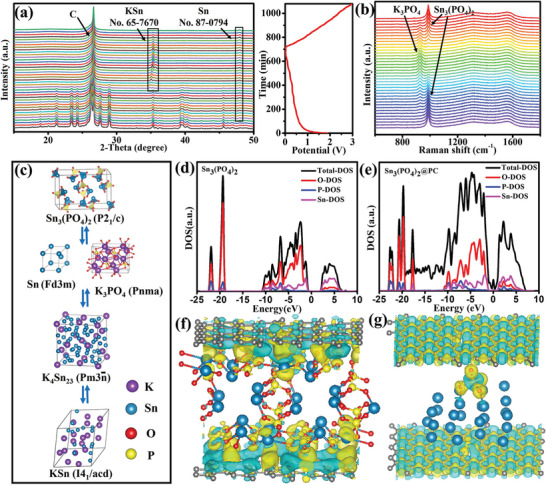
a) In situ XRD patterns of the Sn_3_(PO_4_)_2_@PC‐48 electrode at different charge/discharge states of PIBs. b) In situ Raman spectra of the Sn_3_(PO_4_)_2_@PC‐48 at different charge/discharge states of PIBs. c) The schematic illustration of the reaction mechanism for Sn_3_(PO_4_)_2_@PC upon the potassiation/ depotassiation. Total density of states (DOS) and partial density of states (PDOS) calculated for d) Sn_3_(PO_4_)_2_ and e) Sn_3_(PO_4_)_2_@PC. f) Charge‐density difference of Sn_3_(PO_4_)_2_@PC. g) Charge‐density difference of caused by PO_4_^3−^ in Sn@PC.

To understand the electrochemical reaction mechanism, phase transitions of Sn_3_(PO_4_)_2_ during the first cycle were studied by the combined in situ and ex situ XRD results. **Figure** **5**a shows the in situ XRD patterns of the first cycle. The diffraction peaks at 26.6° can be attributed to conductive carbon paper, which do not alter in intensity and in peak positions upon cycling. Different from the cases of carbon paper, during the discharge process, the diffraction peaks of Sn_3_(PO_4_)_2_ gradually weaken and completely disappeared accompanied by the emergence of Sn. It indicated that the Sn_3_(PO_4_)_2_ was fully reduced. When the cell was discharged to ≈0.8 V, the new peak appeared was assigned to metallic Sn formed from the conversion reaction of Sn_3_(PO_4_)_2_ with K^+^. However, no obvious peak from K_3_PO_4_ was observed, which was likely caused by their poor crystallinity/small size. As the discharge process proceeds, the KSn peaks appears, corresponding to the alloying reaction of Sn with K. Conversely, during the charging process with the increase in the voltage, the peaks of KSn gradually disappeared. A combination of ex situ XRD was used to compensate for the difficulties associated with in situ XRD analysis. As shown in Figure S24 (Supporting Information), during the discharge process, the diffraction peaks of Sn_3_(PO_4_)_2_ gradually weaken (2.2 V) and completely disappeared (0.8 V) accompanied by the emergence of Sn, K_4_Sn_23_, and KSn (0.01 V). It indicated that the Sn_3_(PO_4_)_2_ was fully reduced. Once the Sn_3_(PO_4_)_2_ electrode was discharged to 0.8 V, the XRD signals of Sn_3_(PO_4_)_2_ completely disappeared. However, the signals from Sn were observed, demonstrating the conversion reaction. Once Sn_3_(PO_4_)_2_ electrode was further discharged to 0.5 V, the signals of Sn (JCPDS no. 87‐0794), K_4_Sn_23_ (JCPDS no. 65‐3351), and KSn (JCPDS no. 65‐7670) were emerged, proving the alloying reaction between potassium and Sn. The major peaks of Sn, and the minor peaks of KSn and K_4_Sn_23_ were detected when the electrode discharged to 0.01 V, demonstrating the overlapped conversion and alloying reactions <0.5 V. These coexisted conversions and alloying reactions resulted in a high specific capacity in the voltage range of 0.01–0.5 V. Conversely, in the charging process, with the increase of the voltage up to1.0 V, the peaks of Sn, KSn, and K_4_Sn_23_ gradually disappeared. However, once the electrode was recharged to 1.6 V, the peaks for K_4_Sn_23_ and Sn reemerged. After being fully recharged to 3.0 V, the diffraction peaks assigned to Sn_3_(PO_4_)_2_ were observed, which indicated that the potassiation and depotassiaition of Sn_3_(PO_4_)_2_ might be reversible.

In situ Raman spectroscopy is a useful method to monitor the phase transition of anode during the charging–discharging processes. Figure 5b displayed the in situ Raman spectra during the charging–discharging processes in the first cycle. The Raman peaks at 986 cm^−1^ was ascribed to the symmetric stretching vibration of O—P—O of PO_4_^2−^ in Sn_3_(PO_4_)_2_. The Raman peaks of PO_4_^2−^ blueshift with the decreasing atomic number of metal ions. The peak at 936 cm^−1^ is the characteristic peak of K_3_PO_4_. During the discharge process, this characteristic peak of Sn_3_(PO_4_)_2_ gradually weaken, accompanied by the appearing and increasing of the characteristic peak of K_3_PO_4_. When charged to 3.0 V again, the peak assigned to Sn_3_(PO_4_)_2_ reemerge, which indicated that the transformations during the charge and discharge can be reversible.

The reaction mechanism of the Sn_3_(PO_4_)_2_ electrode was further validated via ex situ TEM/HRTEM images and corresponding SAED patterns. Once the electrode was discharged to 0.01 V, a few lattice fringes with *d*‐spacings of 0.192 nm were observed, which were assigned to the (041) planes of KSn (Figure S25, Supporting Information). Meanwhile, tiny amounts of K_4_Sn_23_ and Sn were still present. This is in agreement with the XRD results. As the electrode was fully discharged to 3.0 V, the HRTEM images illustrated the existence of Sn having lattice spacings of 0.279 and 0.202 nm (Figure S25a,c, Supporting Information).

Based on XRD and HRTEM analyses, the potassium storage mechanism and the reversible reactions of Sn_3_(PO_4_)_2_ during potassiation/depotassiation were established. Accordingly, the potassium storage mechanism could be expressed by the following reversible process:

(4) $${\mathrm{Sn}}_{3}{\left({\mathrm{PO}}_{4}\right)}_{2}+6K^{+}+6e^{-}↔3\mathrm{Sn}+2K_{3}{\mathrm{PO}}_{4}$$

(5) $$23\mathrm{Sn}+4K^{+}+4e^{-}↔K_{4}{\mathrm{Sn}}_{23}$$

(6) $$K_{4}{\mathrm{Sn}}_{23}+19K^{+}+19e^{-}↔23\mathrm{KSn}$$

The working mechanism of Sn_3_(PO_4_)_2_ involving the conversion and alloying reactions upon potassiation was further exhibited in Figure 5c. At first, the Sn_3_(PO_4_)_2_ crystal was converted into Sn and K_3_PO_4_. Subsequently, a cubic K_4_Sn_23_ intermediate phase was formed due to the futher potassiation. At the end of potassiation, the product of KSn was formed. After complete depotassiation, Sn_3_(PO_4_)_2_ was partially recovered along with the intermediate of Sn. To our knowledge, it is the first evidence‐based report on the reaction mechanism of Sn_3_(PO_4_)_2_ acting as the anode of PIBs.

To investigate the effect of morphology for K ion storage, we provide the TEM images of electrodes after 300 cycles (Figure S26, Supporting Information). From the TEM images, the SEI layers of all the electrodes can be clearly observed. The Sn_3_(PO_4_)_2_@PC‐48 exhibits a thin and uniform SEI layer (Figure S26, Supporting Information). The stable SEI formation to the outer surface of the superstructure. It can be seen that the surface is smooth and naked. The quality SEI layer helps maintain the cyclic stability of Sn_3_(PO_4_)_2_@PC‐48. However, small particles can be found on the surface in the SEI layer of Sn_3_(PO_4_)_2_@PC‐24 (Figure S26b, Supporting Information), indicating an inhomogeneous SEI layer. Different from the stable and uniform SEI layer of Sn_3_(PO_4_)_2_@PC‐48, the SEI layer of Sn_3_(PO_4_)_2_@PC‐200 is uneven and broken (Figure S26c, Supporting Information). The re‐formation of SEI led to the continuous decomposition of electrolyte and reducing both the capacity and cycling stability. We provide XPS to further analyze the effect of solid electrolyte interphase (SEI) for K‐ion storage, as shown in Figure S27 (Supporting Information). Figure S27a–c (Supporting Information) shows the high resolution C 1*s* spectra of a) Sn_3_(PO_4_)_2_@PC‐48, b) Sn_3_(PO_4_)_2_@PC‐24, and c) Sn_3_(PO_4_)_2_@PC‐200 after 300 cycles. It can be seen that the SEI film of Sn_3_(PO_4_)_2_@PC‐24 and Sn_3_(PO_4_)_2_@PC‐200 contained abundant organic components (C—O and C=O), demonstrated that the severe decomposition of the solvents in the electrolyte. It would cause the capacity fading. The content of organic components was significantly lower in the SEI of Sn_3_(PO_4_)_2_@PC‐48, implied that the decrease of solvation decomposition. We can distinctly observe that the electrode present different F1*s* XPS spectra in Figure S27d‐f (Supporting Information). The F1*s* XPS spectra could be deconvoluted to distinct peaks at 687.0 and 684.1 eV, which were corresponding to the C—F bond and K—F bond, respectively. From the results of XPS, the K—F content of electrode after cycles in the SEI of Sn_3_(PO_4_)_2_@PC‐48 higher than that of Sn_3_(PO_4_)_2_@PC‐24 and Sn_3_(PO_4_)_2_@PC‐200. Since the enhanced of K—F was beneficial in improving the stability of SEI which prevented the continuous depletion of electrolyte and enhanced the electrode stability for KIBs.

**Figure 6:**
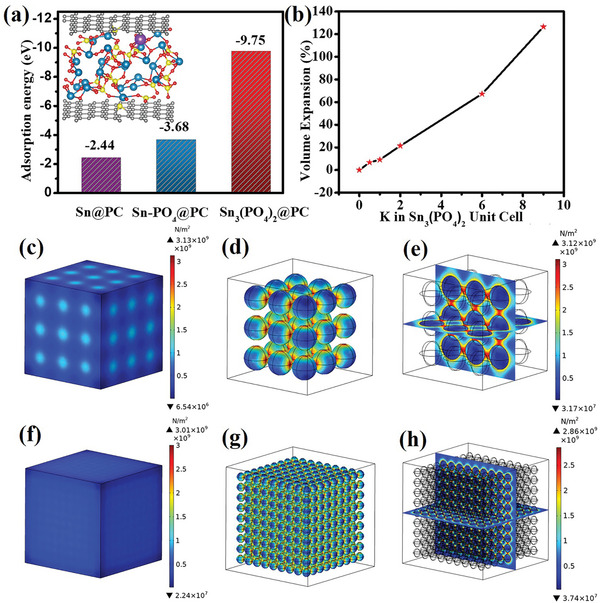
a) Binding energies (Δ*E*_b_) for potassium storage of Sn@PC. b) Calculated volume expansion of Sn_3_(PO_4_)_2_ unit cell after potassiation. c) Overall and d) internal perspective stress contours of Sn_3_(PO_4_)_2_@PC‐200. e) Cross‐sectional stress contours of Sn_3_(PO_4_)_2_ nanoparticles in Sn_3_(PO_4_)_2_@PC‐200. f) Overall and g) internal perspective stress contours of Sn_3_(PO_4_)_2_@PC‐48. h) Cross‐sectional stress contours of Sn_3_(PO_4_)_2_ nanoparticles in Sn_3_(PO_4_)_2_@PC‐48.

To obtain fundamental insight into Sn_3_(PO_4_)_2_@PC on the electronic conductivity and structure stability, DFT calculations were conducted. The optimized model diagrams of Sn_3_(PO_4_)_2_@PC were presented in Figure S28 (Supporting Information). The electronic structures of Sn_3_(PO_4_)_2_ and Sn_3_(PO_4_)_2_@PC were decided by the partial density of states (PDOS) (Figure 5d,e). Sn_3_(PO_4_)_2_@PC possessed a much smaller band gap in comparison to Sn_3_(PO_4_)_2_. This indicated that the heterostructure changed the electron distribution and contributed to the movement of the conduction band toward the Fermi level. As shown in the charge density difference plot (Figure 5f), the obvious charge transformation from Sn_3_(PO_4_)_2_ to PC in the Sn_3_(PO_4_)_2_@PC heterostructure resulted in a firm and intimate contact while improving the structure stability. Moreover, the introduction of PO_4_^3−^ in the Sn@PC heterostructure (Sn‐PO_4_@PC) was helpful to increase the electron injection (Figure 5g). It also induced a charge transfer to form positively and negatively charged surfaces, which was favorable to improving the adsorption and transfer kinetics of K^+^. The binding energies of K ions (Δ*E*_b_) in Sn@PC, Sn‐PO_4_@PC, and Sn_3_(PO_4_)_2_@PC were also calculated for comparison. Figure S28 (Supporting Information) presented the structure models of Sn@PC, Sn‐PO_4_@PC and Sn_3_(PO_4_)_2_@PC during the potassiation and depotassiation states. Sn_3_(PO_4_)_2_@PC possessed the lowest binding energy (−9.75 eV), smaller than those of Sn‐PO_4_@PC (−3.68 eV), and Sn@PC (−2.44 eV). It suggested that the Sn_3_(PO_4_)_2_@PC was the most energetically favorable for K adsorption (**Figure** **6**a). The optimized electronic structure and strong K binding ability for Sn_3_(PO_4_)_2_@PC accounted for its fast and stable K storage performance.

To understand the correlation between the structural variation and mechanical properties, the volume expansion and structure stress after ion intercalation was simulated by DFT calculation and FEA analysis. Detailed information regarding the simulation parameters was provided in the Supporting Information. It was obvious that the Sn_3_(PO_4_)_2_ had undergone a huge volume expansion (≈120%) after embedding potassium atoms (Figure 6b). Therefore, the mechanical strength of the Sn_3_(PO_4_)_2_@PC superstructures was further analyzed by FEA.

Different models (Sn_3_(PO_4_)_2_@PC‐48 and Sn_3_(PO_4_)_2_@PC‐200) were established to demonstrate the stress distribution after 120% volume expansion by FEA. Figure 6c–h displayed the stress contours and the corresponding stress colormaps of different models. For Sn_3_(PO_4_)_2_@PC‐200, the stress concentration distribution gradually increased from the inner to the outer of Sn_3_(PO_4_)_2_ nanoparticles (Figure 6c–e; Figure S29, Supporting Information). A significant stress region with a maximum stress value of 3.13 GPa was produced on the surface contact area between the carbon and the Sn_3_(PO_4_)_2_ nanoparticles. Compared to Sn_3_(PO_4_)_2_@PC‐200, the Sn_3_(PO_4_)_2_@PC‐48 possessed maximum stress of 3.01 GPa. The stress concentrated area was distributed on its contact surface (Figure 6f,j,h; Figure S29b, Supporting Information), which indicated the size‐dependent electro‐chemomechanical behavior. Reducing the particle size of Sn_3_(PO_4_)_2_ in Sn_3_(PO_4_)_2_@PC superstructure was favorable for releasing the stress and improving cycle stability. Based on the above analysis, the unique morphology and ultrathin particle size in the superstructure played a vital role in stress release and enhancing its lifespan.

In view of the excellent performances of Sn_3_(PO_4_)_2_@PC‐48 in half cells, it is tested in full cells by using homemade prussian blue (PB) as cathode material (Figure S30, Supporting Information). The full cell was tested within 0.8–3.2 V at density of 0.5 A g^−1^_anode_. As shown in Figure S30 (Supporting Information), the full cell displays a very stable cycling. After 100 cycles at 0.5 A g^−1^, the capacity is still 71.5 mA h g^−1^, corresponding to a capacity retention of 92.8%.

**Figure 7:**
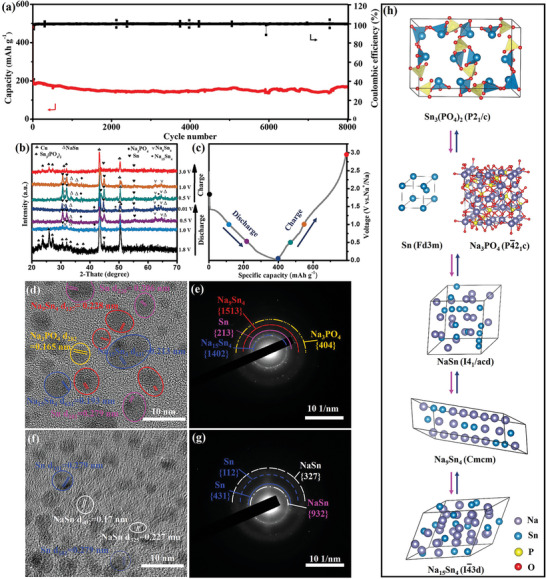
a) Long‐term cycling performance of Sn_3_(PO_4_)_2_@PC‐48 at a current density of 5 A g^−1^ in SIBs. b,c) Ex situ XRD patterns of the Sn_3_(PO_4_)_2_@PC‐48 electrode at different charge/discharge states of SIBs. d) TEM image and e) SAED pattern of the fully discharged Sn_3_(PO_4_)_2_@PC‐48 electrode in SIBs. f) TEM image and g) SAED pattern of the fully charged Sn_3_(PO_4_)_2_@PC‐48 electrode in SIBs. h) The schematic illustration of the reaction mechanism for Sn_3_(PO_4_)_2_@PC of sodium insertion/ extraction.

Inspired by the remarkable performance of Sn_3_(PO_4_)_2_@PC‐48 electrode in PIBs, the Na^+^ storage performance and mechanism of Sn_3_(PO_4_)_2_@PC‐48 were further evaluated. As shown in **Figure** **7**a, for SIBs, the Sn_3_(PO_4_)_2_@PC‐48 delivered 96.0% capacity retain after 8000 cycles, with a capacity of 202.5 mAh g^−1^ at 5A g^−1^, which exceeds those of most reported anodes for SIBs (Table S2, Supporting Information). The CE stabilized at ≈99.69% for 5 A g^−1^ throughout the cycles. In contrast, the cycling stabilities of Sn_3_(PO_4_)_2_@PC‐24 and Sn_3_(PO_4_)_2_@PC‐200 for sodium storage were inferior compared to Sn_3_(PO_4_)_2_@PC‐48 (Figures S37 and S38, Supporting Information). The capacity retention of Sn_3_(PO_4_)_2_@PC‐24 and Sn_3_(PO_4_)_2_@PC‐200 were only 65.8% and 19.9% after 2000 cycles. The excellent long‐term reversibility of Sn_3_(PO_4_)_2_@PC‐48 indicated that the unique superstructure favored the stability of the electrode during cycling. The stable superstructure can effectively resolve the critical issues of severe volume expansion and poor conductivity. The superior Na storage of the Sn_3_(PO_4_)_2_‐based electrodes was achieved by optimizing the structure and the conductive framework. The Na^+^ storage mechanisms of the Sn_3_(PO_4_)_2_ anode during charge and discharge were investigated by ex situ XRD (Figure 7b,c) and ex situ HRTEM images/SAED (Figure 7d–g). As shown in Figure 7b,c, during the discharge process, the diffraction peaks of Sn_3_(PO_4_)_2_ gradually weaken (1.8 V) and completely disappeared (0.5 V) accompanied by the emergence of Na_3_PO_4_, Sn, NaSn, Na_9_Sn_4_, and Na_15_Sn_4_ (0.01 V). This indicated that the Sn_3_(PO_4_)_2_ was fully reduced. The HRTEM analysis was performed to confirm the formation of Sn, NaSn, Na_9_Sn_4_, and Na_15_Sn_4_ during the full discharge state (Figure 7d,e). During the subsequent charging process, the diffraction peaks of Sn, NaSn, Na_9_Sn_4_, and Na_15_Sn_4_ gradually weaken, whereas the phases of Sn_3_(PO_4_)_2_ were again found (3.0 V). HRTEM and SAED images at full charge state indicated the high reversibility of the Na storage reaction (Figure 7f,g). However, the peaks of Na_3_PO_4_ and Sn did not completely disappear and hence, proved that the capacity was attenuated at the beginning. The ex situ characterization outcome showed that the Na^+^ storage mechanism of Sn_3_(PO_4_)_2_ was different from K^+^. In addition, the conversion reaction mechanism was comparatively complex in Na‐ion systems. The electrochemical reactions upon cycling were shown in Figure 7h and could be described by Equations (7), (8), and 9.

(7) $${\mathrm{Sn}}_{3}{\left({\mathrm{PO}}_{4}\right)}_{2}+3{\mathrm{Na}}^{+}+3e^{-}↔\mathrm{Sn}+{\mathrm{Na}}_{3}{\mathrm{PO}}_{4}$$

(8) $$\mathrm{Sn}+{\mathrm{Na}}^{+}+e^{-}↔\mathrm{NaSn}$$

(9) $$4\mathrm{NaSn}+11{\mathrm{Na}}^{+}+11e^{-}↔{\mathrm{Na}}_{15}{\mathrm{Sn}}_{4}$$

We assembled full cells using Sn_3_(PO_4_)_2_@PC‐48 as anode and homemade sodium Prussian blue analog as the cathode. The full cell was tested within 0.8–3.2 V at a current density of 0.5 A g^−1^_anode_. As shown in Figure S39 (Supporting Information), the full cell exhibited a good stability. After 80 cycles at 0.5 A g^−1^, the capacity was still 117.4 mA h g^−1^_anode_. The good stability was benefitting from the well‐designed superstructure of Sn_3_(PO_4_)_2_@PC‐48, and the structure was not cracked and there was no excessive defect induced during the repeated cycling.

## 3 Conclusion

Inspired by the controllable CPA growing process of Sn‐based phosphate MOFs, we had constructed an Sn‐MOF superstructure derived Sn_3_(PO_4_)_2_ nanocrystal confined in the interlinked carbon framework. Owing to the synergistic effect between the unique superstructure and the excellent intrinsic activity of Sn_3_(PO_4_)_2_, the Sn_3_(PO_4_)_2_@PC‐48 presented high capacity and outstanding cycle stability for PIBs and SIBs (324 mAh g^−1^ at 0.1 A g^−1^ after 300 cycles). Even after 10000 cycles at 5 A g^−1^ the capacity retention remained at ≈90.1% for PIBs and a reversible capacity of 202.5 mAh g^−1^ at 5A g^−1^ with 96.0% capacity retain after 8000 cycles for SIBs. It is one of the best performances among the anodes for K^+^/ Na^+^ storage reported so far. Systematic physicochemical/structural analysis combined with DFT calculation and FEA revealed that the intrinsic property of Sn_3_(PO_4_)_2_@PC and its unique superstructure cooperated to boost the K^+^ and Na^+^ storage kinetics and performance. The reason was attributed to the promoted electronic conductivity, enhanced K binding ability and improved structure stabilization. The maximum stress significantly decreased with the size reduction of Sn_3_(PO_4_)_2_ nanoparticles in Sn_3_(PO_4_)_2_@PC superstructure, which significantly reduced the risk of surface cracks and particle pulverization during potassiation/ sodiation. The new findings could open the door for exploring more innovations of microstructures and functionalities for applications in energy storage and other fields by control the crystallization process in MOFs.

## Conflict of Interest

The authors declare no conflict of interest.

## Supporting information

Supporting Information

## Data Availability

The data that support the findings of this study are available from the corresponding author upon reasonable request.
