# Supplementary material for: Stress‐Dispersed Superstructure of Sn3(PO4)2@PC Derived from Programmable Assembly of Metal–Organic Framework as Long‐Life Potassium/Sodium‐Ion Batteries Anodes
Source: Adv Sci (Weinh). 2023 Apr 23;10(17):2206587. doi: 10.1002/advs.202206587 (PMC10265062; doi:10.1002/advs.202206587)
Supplement: Supplementary file 1 — Supporting Information [file ADVS-10-2206587-s001.pdf]

ADVANCED SCIENCE
Open Access

Supporting Information

for *Adv. Sci.*, DOI 10.1002/advs.202206587

Stress-Dispersed Superstructure of $Sn_3(PO_4)_2$@PC Derived from Programmable Assembly of Metal–Organic Framework as Long-Life Potassium/Sodium-Ion Batteries Anodes

Huimin Jiang, Shuo Zhang, Liting Yan, Yanlong Xing, Zhichao Zhang, Qiuju Zheng, Jianxing Shen, Xuebo Zhao* and Lianzhou Wang**

Supporting Information

Stress-Dispersed Superstructure of $Sn_3(PO_4)_2$@PC Derived from Programmable Assembly of Metal-organic Framework as Long-life Potassium/Sodium-Ion Batteries Anodes

Huimin Jiang[1, 3]#, Shuo Zhang[1, 3]#, Liting Yan[1], Yanlong Xing[4], Zhichao Zhang[5], Qiuju Zheng[1], Jianxing Shen[1], Xuebo Zhao[1, 3]*, Lianzhou Wang[2]**

1. Huimin Jiang, Liting Yan, Shuo Zhang, Qiuju Zheng, Jianxing Shen, Xuebo Zhao

School of Materials Science and Engineering, Qilu University of Technology (Shandong Academy of Sciences), 250353, Jinan, PR China

E-mail: (yanlt@qlu.edu.cn; zhaoxuebo@upc.edu.cn)

2. Lianzhou Wang

School of Chemical Engineering and Australian Institute for Bioengineering and Nanotechnology, University of Queensland, St Lucia, QLD 4072, Australia.

E-mail: (l.wang@uq.edu.au)

3. Huimin Jiang, Shuo Zhang, Xuebo Zhao

State Key Laboratory of Heavy Oil Processing, College of Chemistry and Chemical Engineering, China University of Petroleum (East China), 266580, Qingdao, PR China

4. Yanlong Xing

Key Laboratory of Emergency and Trauma, Ministry of Education, Hainan Medical University, 571199, Haikou, PR China

5. Zhichao Zhang

Tianmu Lake Institute of Advanced Energy Storage Technologies Co., Ltd., 213300, Liyang, PR China

[#] H. Jiang. and S. Zhang. contributed equally to this work

Experimental section

Synthesis of p-xylylenediphosphonic acid (H_4xdp)[1]: The ligand was synthesized by reacting alpha, alpha'-dibromo-p-xylene with triethyl phosphite and followed by refluxing the obtained oil with conc. hydrochloric acid according to the literature method. Block colorless crystals were obtained from the water solution by slow evaporation.

Synthesis of Sn-MOF[1]: Sn-MOF precursor was prepared according to previous work. In a typical procedure, SnC_2O_4 (0.240 g, 1.0 mmol) was stirred together with p-xylylenediphosphonic acid (H_4xdp) (0.140 g, 0.5 mmol) in 16ml deionised water. The acidified solution was then placed in a 25 cm^3 Ace pressure tube and heated at 180 °C for 12-200 h. The resultant white crystalline material was thoroughly washed with deionised water several times and dried at 80 ^oC for 12 h under vacuum.

Synthesis of prussian blue products: The synthesis of the PB samples were according to previously reported methods[2]. In particular, 1 mmol $K_4Fe(CN)_6$ or $Na_4Fe(CN)_6$ was dissolved into 160 mL deionized water to form A solution, and 2 mmol $FeCl_3$ was dissolved into 40 mL deionized water to form B solution. Then, the B solution is added dropwise to the A solution. The mixed solution was continued to

be stirred for 2h and then aged for 24 h after the A and B solutions are evenly mixed. Finally, the aged dark blue precipitate is centrifuged to obtain the PB products.

Preparation of $Sn_3(PO_4)_2$@PC: In a typical procedure, 500 mg Sn-MOF precursor was placed in a porcelain boat. Then, the boat was heated at 630 ^oC under a constant flow of argon at 30 mL·min^{-1} for 120 min with the heating rate of 20 ^oC min^{-1}. The final black powder was collected when the temperature dropped to room temperature under argon atmosphere.

Characterization

The crystal structure of sample was characterized by powder X-ray diffraction (XRD) (PANalytical Inc.) using Cu Kα irradiation operating at 45 KV and 40 mA with a fixed slit. Morphology of sample was observed by a JEOL JSM-7500F (Japan) Field Emission Scanning Electron Microscopy (FESEM). TEM (HRTEM) images were measured using a JEOL JEM2100F (Japan) Transmission Electron Microscope for investigating the information on lattice and fringe. X-ray photoelectron spectroscopy (XPS) analyses were performed with a Thermo ESCALAB 250 (USA) spectrometer using an Al K_α (1486.6 eV) photon source. Raman spectrum was recorded using JY HR800 under ambient conditions.

Electrochemical Measurements.

K and Na storage properties of $Sn_3(PO_4)_2$@PC-24, $Sn_3(PO_4)_2$@PC-48 and $Sn_3(PO_4)_2$@PC-200 were achieved by using CR2032 coin cells. Active materials, super P, and CMC (sodium carboxymethyl cellulose, MW = 250 000) with a mass ratio of 7:1.5:1.5 were mixed to make working electrodes with an average mass

loading of 1.0 mg cm^{-2}. For the assembling of coin cells, sodium and potassium foil, glass microfibre filters (whatman), 1 M $NaClO_4$ and 1 M KFSI in ethylene carbonate (EC), dimethyl carbonate (DMC), and ethyl methyl carbonate (EMC) (1:1:1, volume ratio) were employed as counter, separator, and electrolyte, respectively. The electrochemical properties were measured in the range of 0.01-3 V on a battery test system (LAND CT-2001A). The cyclic voltammetry (CV) measurements and electrochemical impedance spectroscopy (EIS) were carried out on an electrochemistry workstation (CHI760E, China). EIS measurements were carried out using a perturbation voltage of 5 mV in a range of 10^{-2}-10^{5} Hz. CV curves were measured over the range from 0.1 to 1.0 mV s^{-1} between 0.01 and 3 V. The cathode electrodes were fabricated by dispersing the PB, super P, and PVDF with a weight ratio of 7:1.5:1.5. The cathode collector was Al foils and the following steps were similar to the anode electrodes.The $Sn_3(PO_4)_2$@PC-48 anode and the prussian blue cathodes were pre-potassiation for 5 cycles before the full cells were assembled.

DFT Calculations. Density Functional Theory (DFT) calculations were carried out by the Vienna Ab initio Simulation Package. Generalized gradient approximation (GGA) of Perdew-Burke-Ernzerhof (PBE) was employed to calculate the exchange-correlation function with a plane wave cutoff energy of 500 eV. The electronic energy was considered self-consistent when the energy change was smaller than 10^{-5} eV. A geometry optimization was considered convergent when the force change was smaller than 0.02 eV/Å. Furthermore, Grimme's DFT-D2 methodology was used to describe the dispersion interactions. The k-point mesh is sampled by a

separation of 0.05 Å^{-1}. In addition, the density of states (DOS) was investigated when a 3 × 5 × 1 Monkhorst-Pack scheme k-point mesh for the structures was used.

The binding energies of K ions (ΔE) in Sn@PC, Sn-PO_4@PC and $Sn_3(PO_4)_2$@PC were also calculated for comparison, according to the following equation:

$$\Delta E = E_{\mathrm{K-Sn_3(PO_4)_2@PC}} - E_{\mathrm{Sn_3(PO_4)_2@PC}} - E_{\mathrm{K}}$$

Where $E_{\mathrm{K-Sn_3(PO_4)_2@PC}}$, $E_{\mathrm{K-Sn_3(PO_4)_2@PC}}$, and E_K correspond to the energies of K binding $Sn_3(PO_4)_2$@PC, $Sn_3(PO_4)_2$@PC and K ion, respectively.

Finite element method. The finite element method of the stress distribution in the $Sn_3(PO_4)_2$@PC is operated by COMSOL Multiphysics 5.6. The key scheme is to simulate the stress intensity under the same volume expansion. In this case, the particle diameters of $Sn_3(PO_4)_2$ in $Sn_3(PO_4)_2$@PC-48 and $Sn_3(PO_4)_2$@PC-200 are 10 nm and 30 nm, respectively. In actual modeling, the total volume of nanoparticles in different models were same, and the particle position was uniform distribution. The related parameters, density (ρ), young's modulus(E), are list in Table.S1.

Table S1. Material properties in FE simulations.

Phases	Young's modulus/GPa	Poisson's ration	Density/g cm^{-3}
Sn	50	0.36	5.796
C	1	0.3	2.3

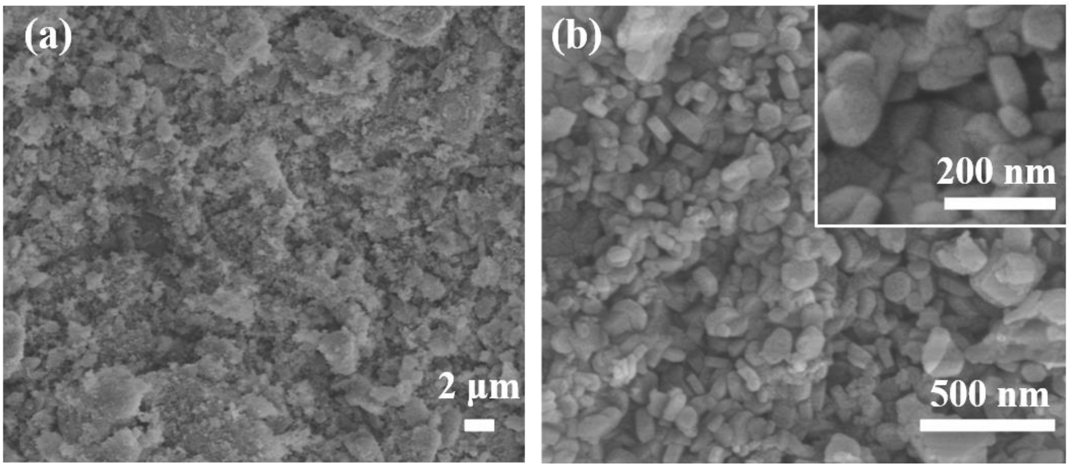

Fig. S1. a) and b) FESEM images of Sn-MOF-24.

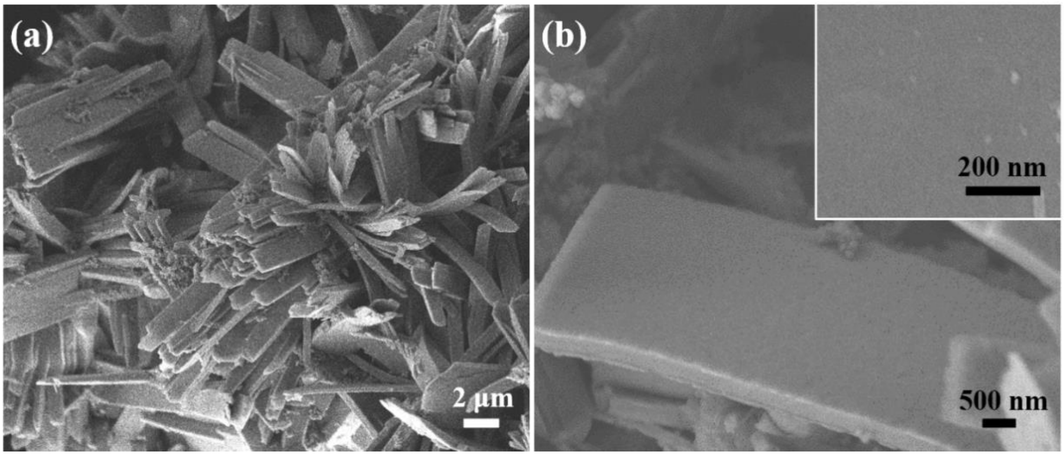

Fig. S2. a）FESEM images of Sn-MOF-200. b) The surface of Sn-MOF-200.

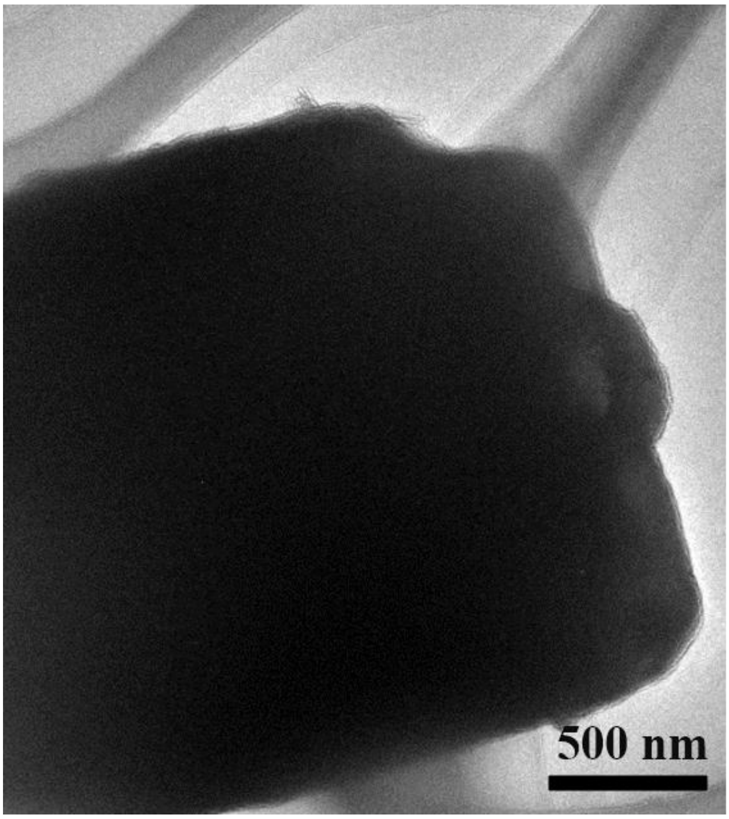

Fig. S3. TEM images of Sn-MOF-200.

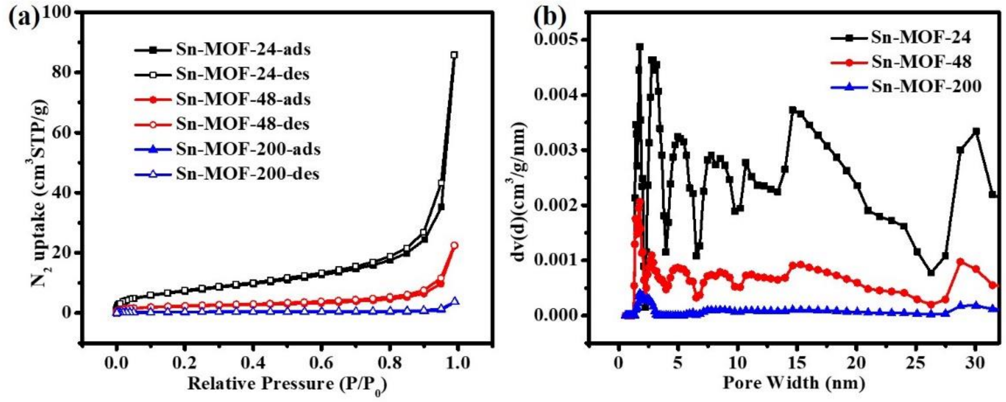

Fig. S4. (a) N_2 sorption isotherms at 77 K for Sn-MOF. (b) Pore size distribution of Sn-MOFs.

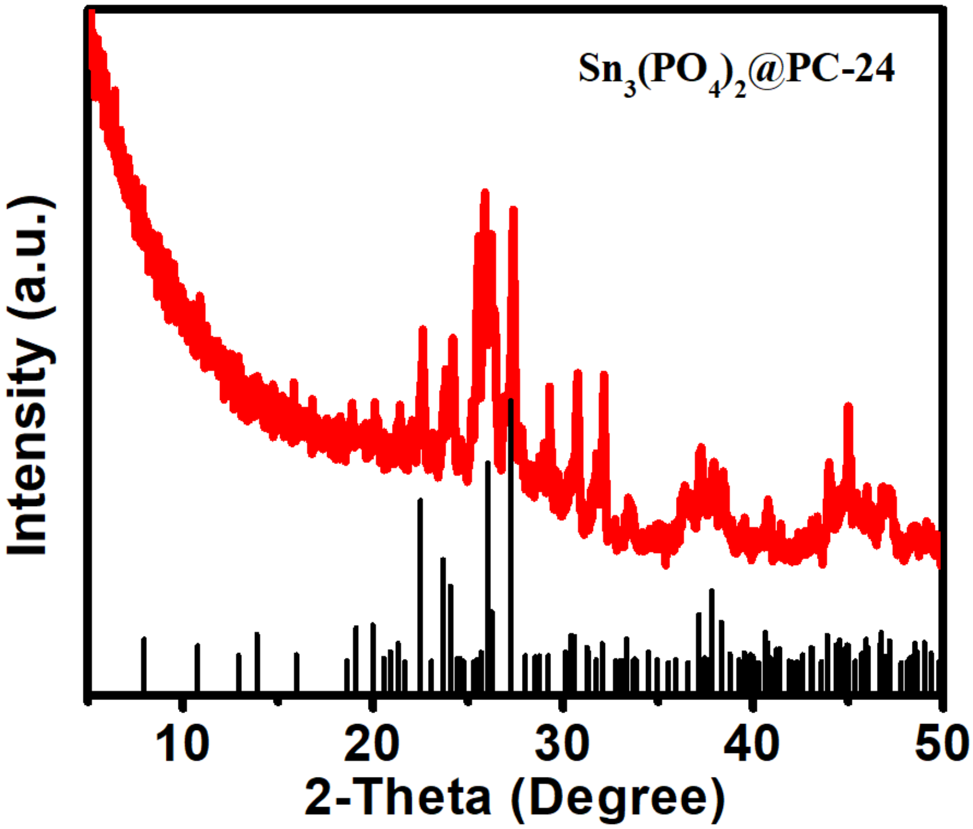

Fig. S5. XRD pattern of $Sn_3(PO_4)_2$@PC-24.

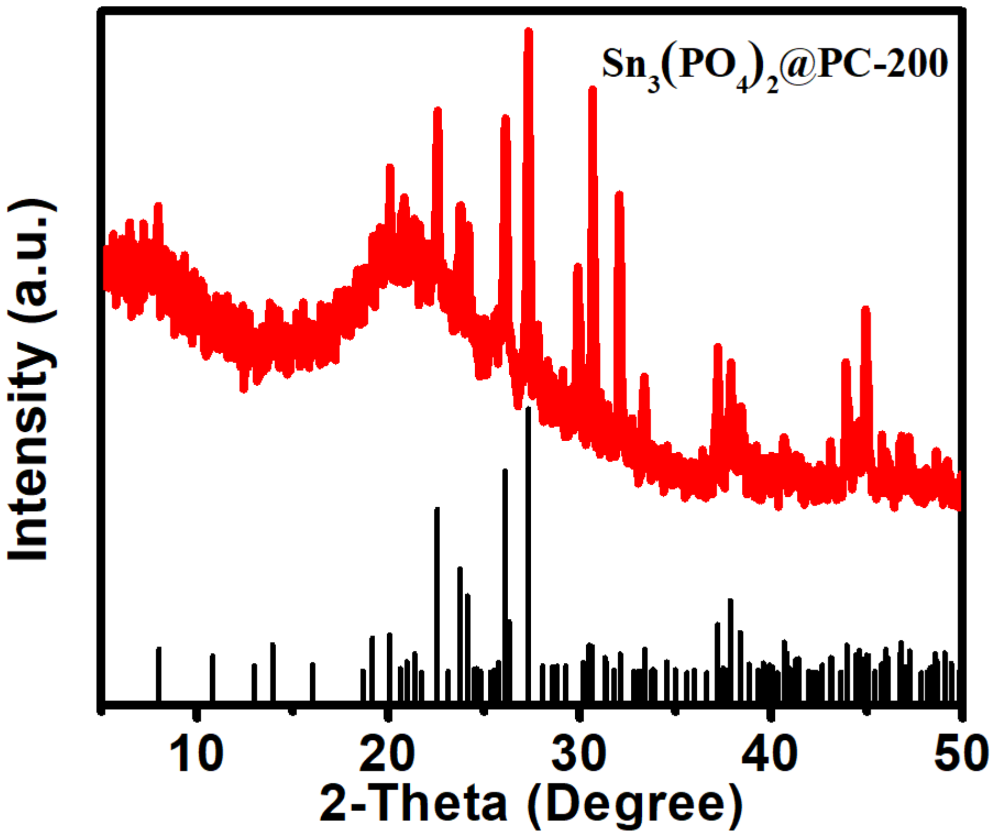

Fig. S6. XRD pattern of $Sn_3(PO_4)_2$@PC-200.

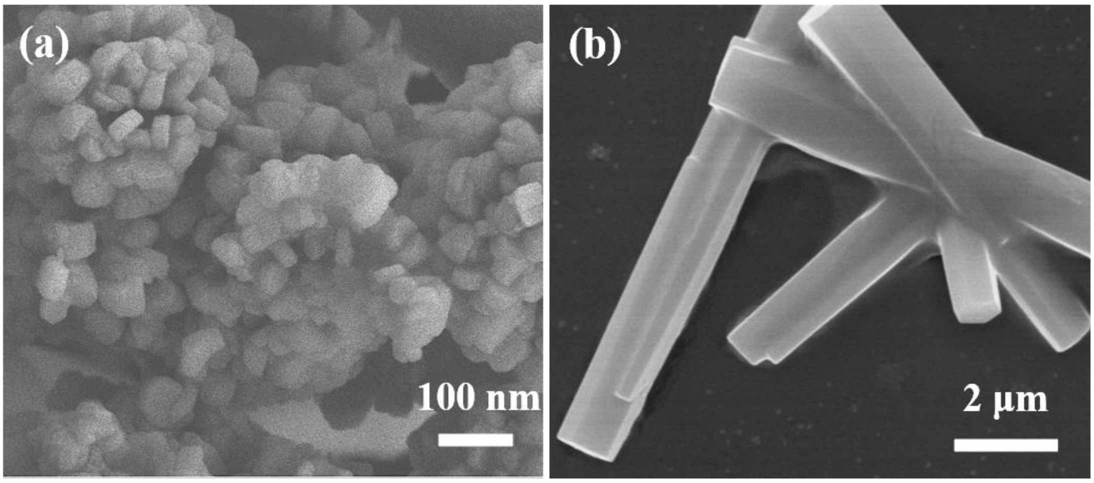

Fig. S7. FESEM images of a) $Sn_3(PO_4)_2$@PC-24 and b) $Sn_3(PO_4)_2$@PC-200.

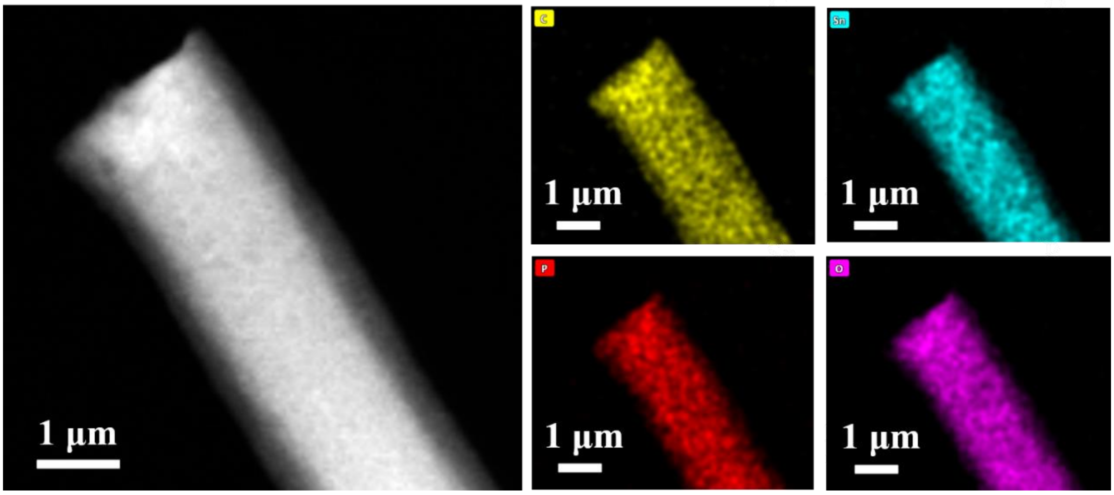

Fig. S8. TEM-EDS mapping of the $Sn_3(PO_4)_2$@PC-48.

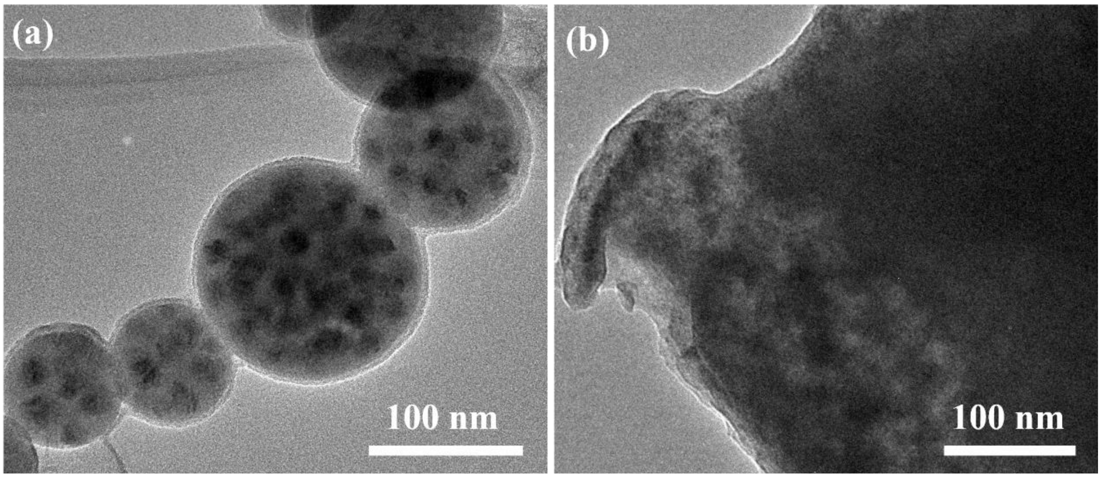

Fig. S9. TEM images of a) $Sn_3(PO_4)_2$@PC-24 and b)$Sn_3(PO_4)_2$@PC-200.

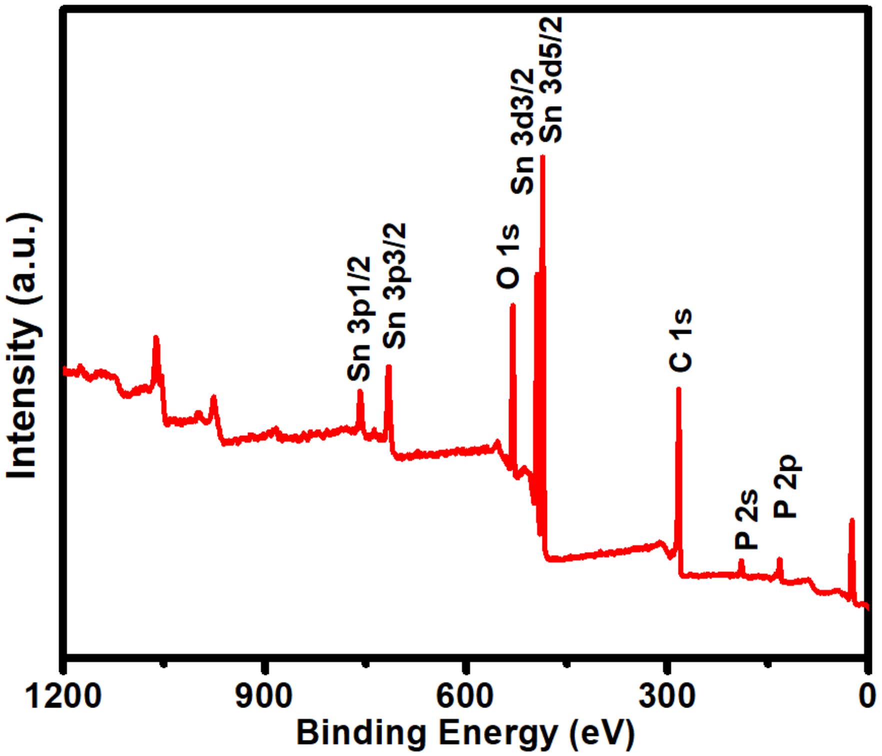

Fig. S10. X-ray photoelectron spectroscopy of $Sn_3(PO_4)_2$@PC-48.

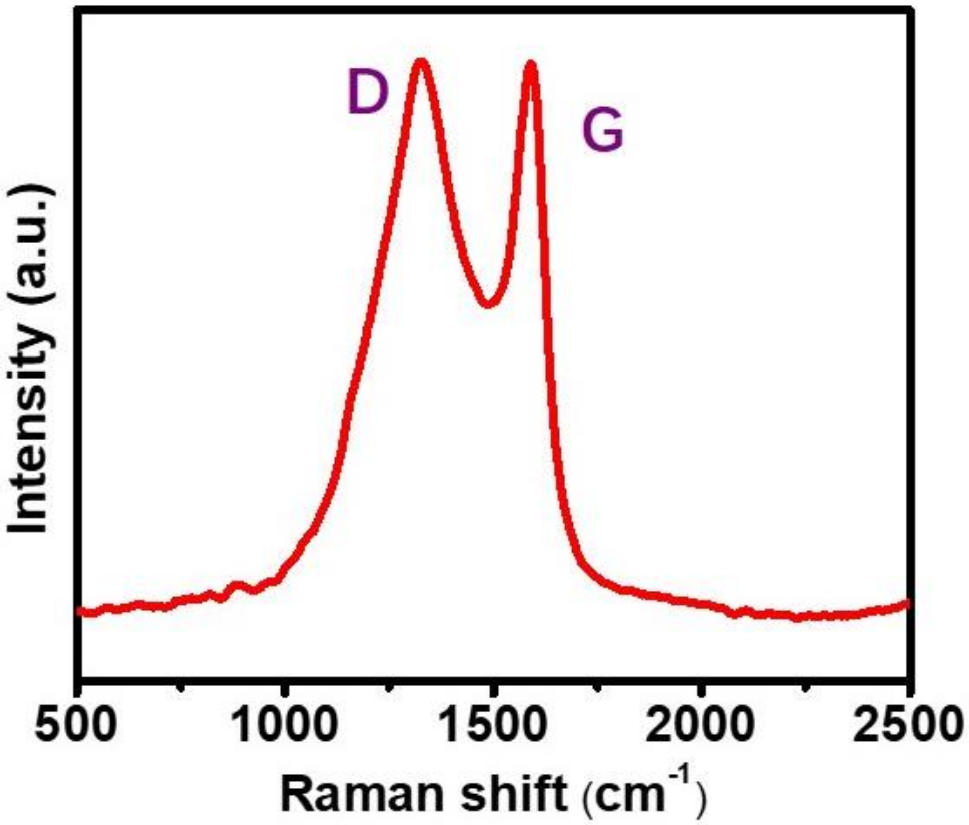

Fig. S11. Raman spectrum of $Sn_3(PO_4)_2$@PC-48.

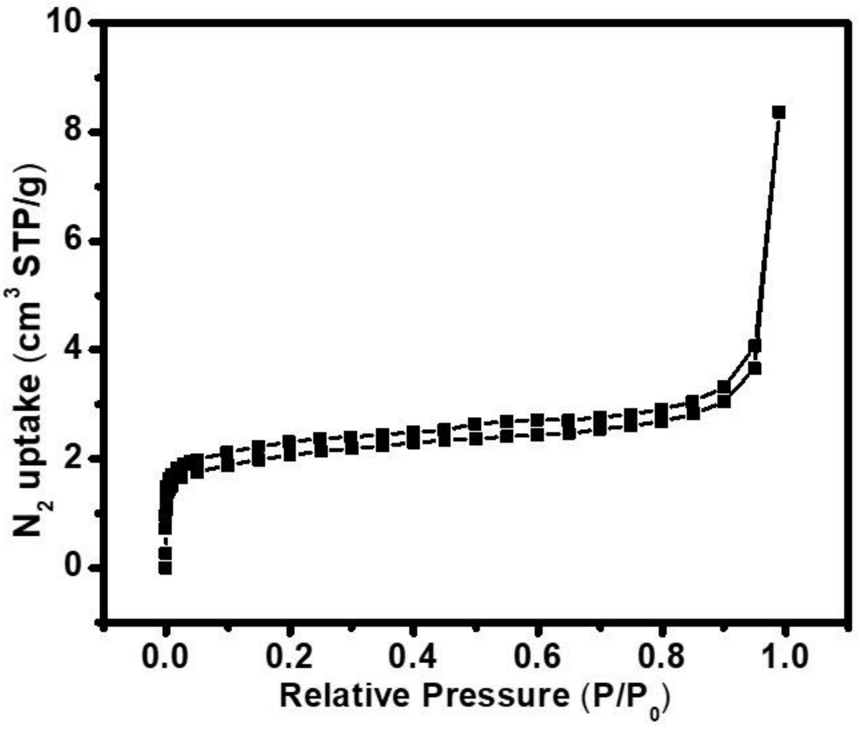

Fig. S12. N_2 sorption isotherms of $Sn_3(PO_4)_2$@PC-48.

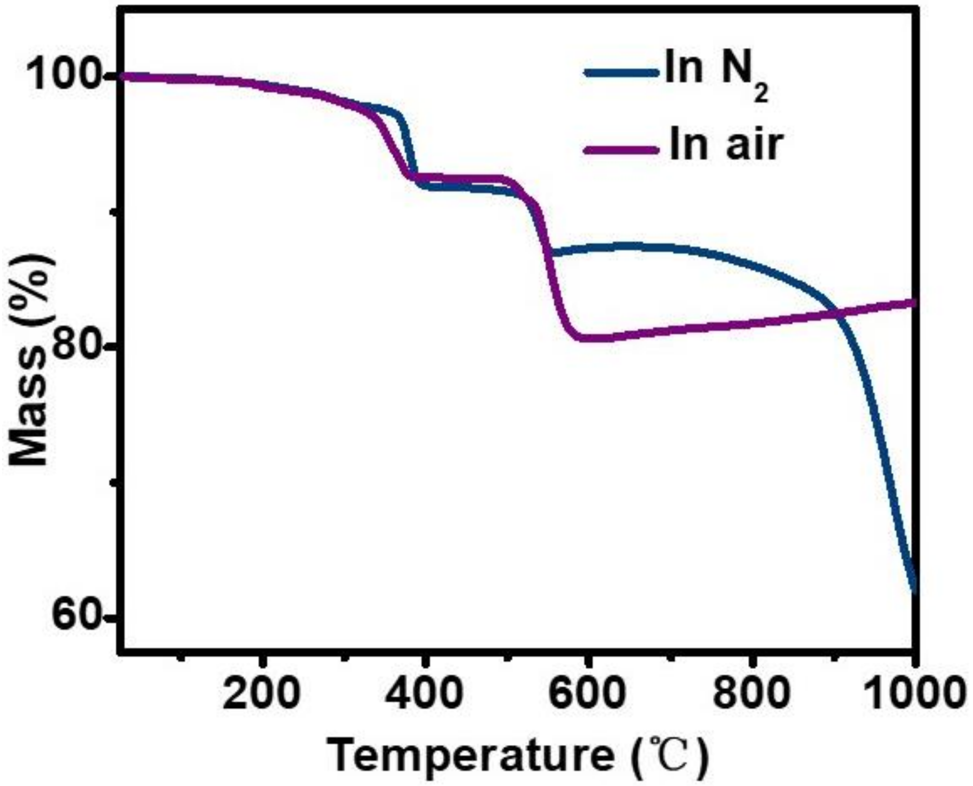

Fig. S13. Thermogravimetric analysis of Sn-MOF-48 in air and N_2.

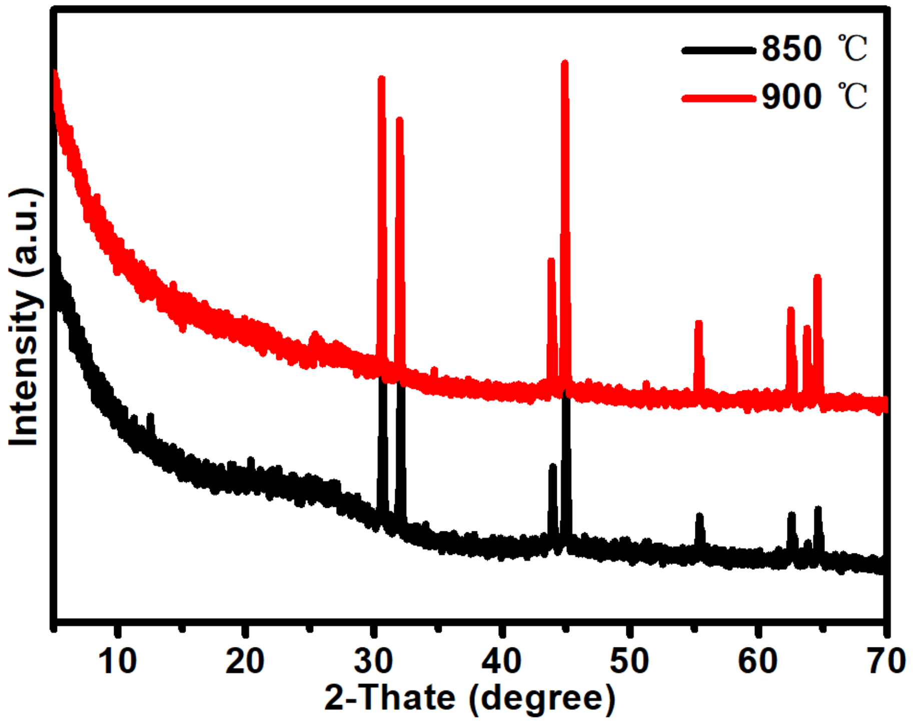

Fig. S14. XRD patterns of Sn-MOF-48 calcined at different temperature.

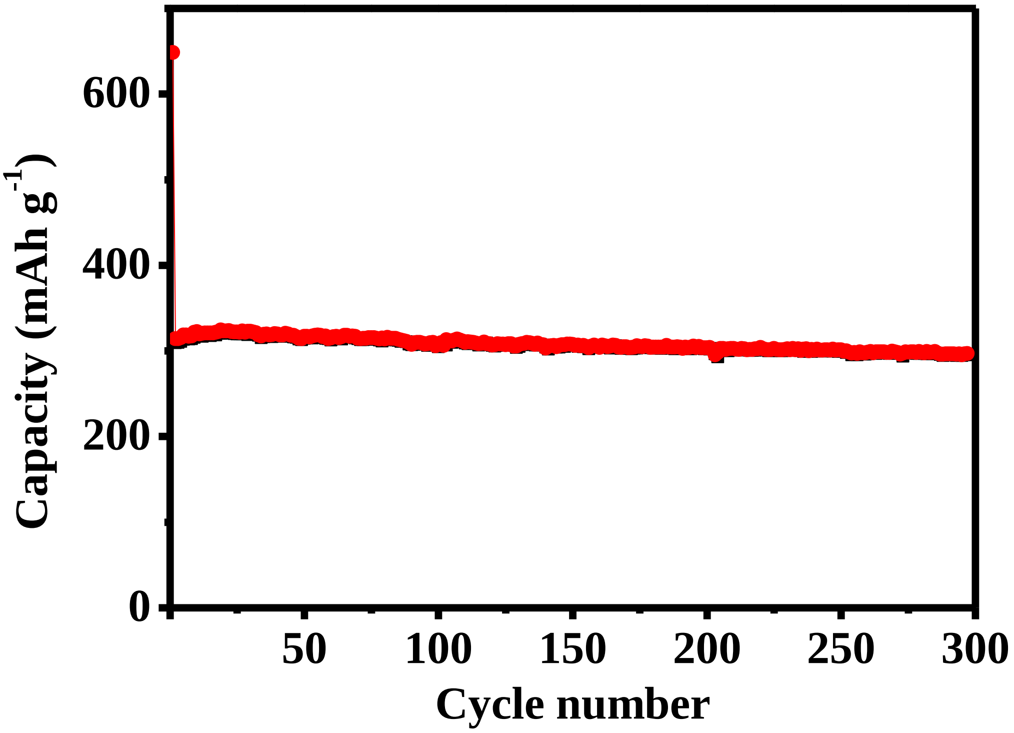

Fig. S15. Cycle performance of the $Sn_3(PO_4)_2$@PC-48 electrodes for K ion storage at 0.1 A g^{-1} of higher mass loading.

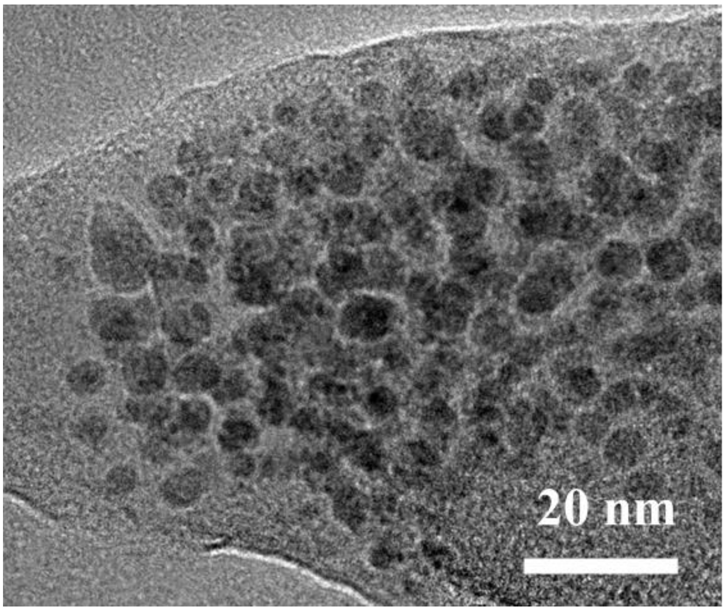

Fig. S16. TEM images of $Sn_3(PO_4)_2$@PC-48 after 300 cycles.

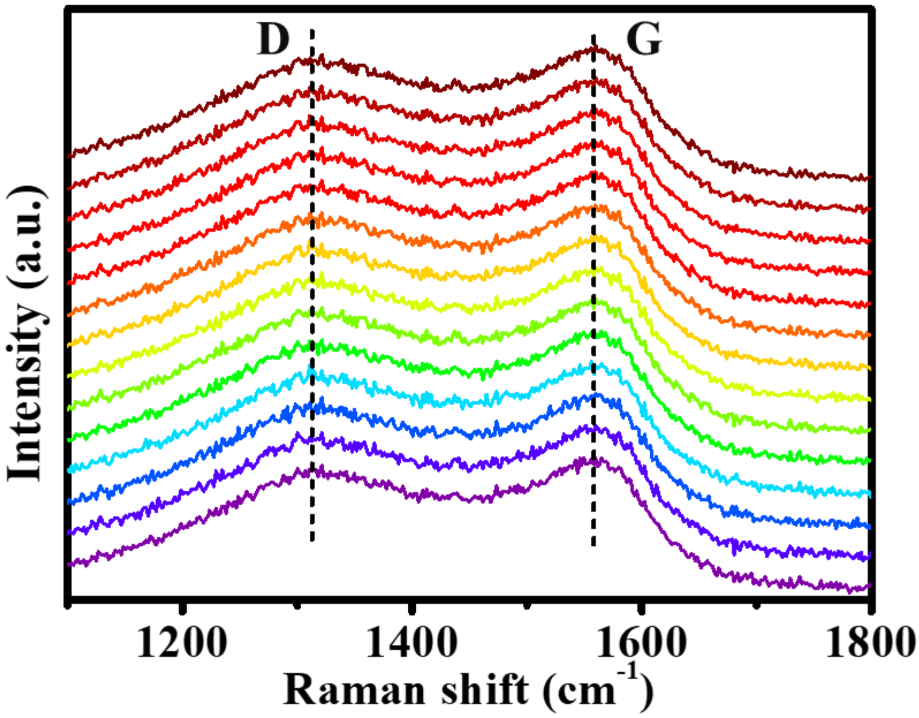

Fig. S17. In situ Raman spectra of the $Sn_3(PO_4)_2$@PC-48 at different charge/discharge states of PIBs.

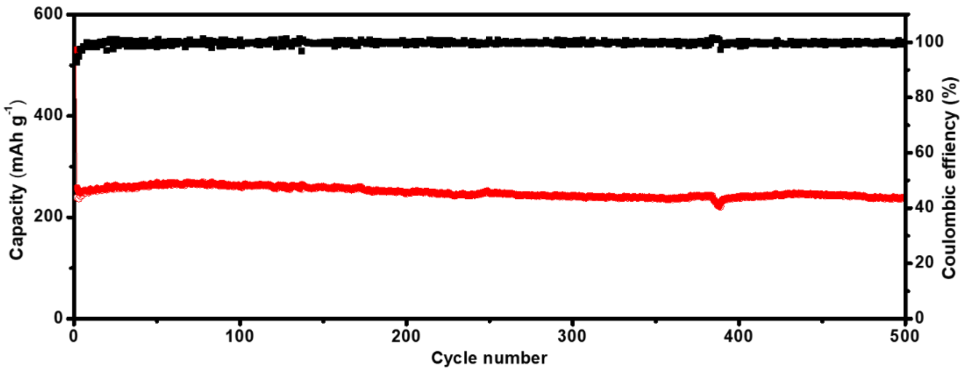

Fig. S18. Cycle performance of the $Sn_3(PO_4)_2$@PC-48 electrodes for K ion storage at 0.5 A g^{-1}.

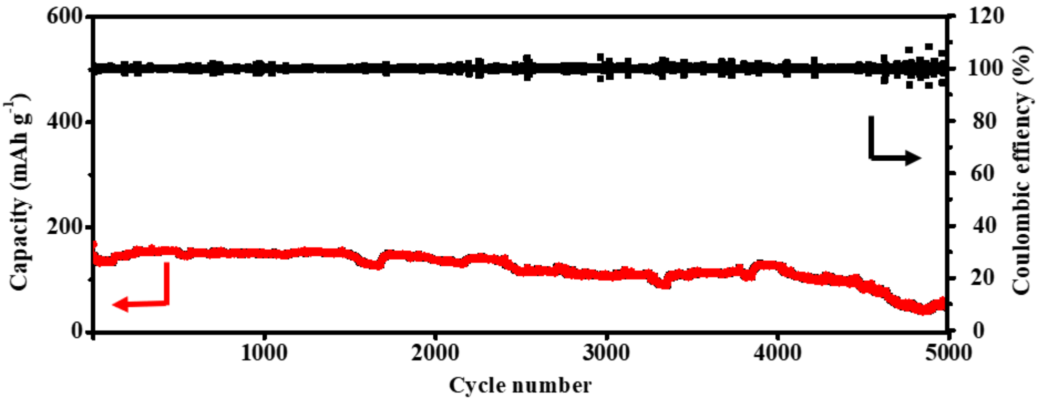

Fig. S19. Cycle performance of the $Sn_3(PO_4)_2$@PC-24 electrodes for K ion storage at 5 A g^{-1}.

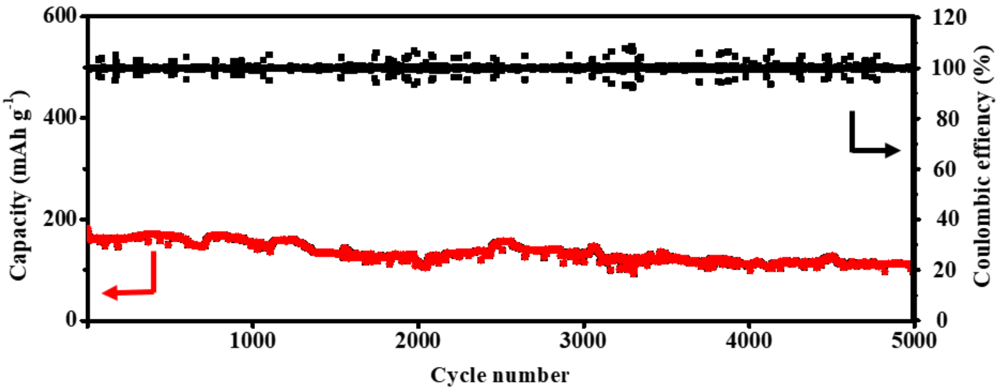

Fig. S20. Cycle performance of the $Sn_3(PO_4)_2$@PC-200 electrodes for K ion storage at 5 A g^{-1}.

Table. S2 Comparison of cycling performance of $Sn_3(PO_4)_2$@PC and the reported anodes for PIBs and SIBs

**Anode material**	**Reversible capacity (mAh g^{-1})**	**Cycle number**	**Current density (mA g^{-1})**	**Battery type**	**Reference**
**$Sn_3(PO_4)_2$@PC-48**	**324**<br>**144**	**300**<br>**10000**	**100**<br>**5000**	**PIBs**	**This work**
Few layered graphene microspheres	252<br>230	100<br>1000	100<br>200	PIBs	3
SnO_2@CF	231.7	400	1000	PIBs	4
VN@CFs-550	232<br>133.2	800<br>1600	50<br>2000	PIBs	5
o-P-$CoTe_2$/MXene	373.7<br>232.3	200<br>2000	200<br>2000	PIBs	6
$CoSe_2$/$FeSe_2$@C-II	271.4	300	2000	PIBs	7
APC-700	100.3	10000	5000	PIBs	8
**$Sn_3(PO_4)_2$@PC-48**	**372.8**<br>**202.5**	**100**<br>**8000**	**100**<br>**5000**	**SIBs**	**This work**
MPC-1000	145	5000	5000	SIBs	9
DS-Cu_3P	325	100	50	SIBs	10
Co_3O_4@C	166	1700	5000	SIBs	11
WS_2/ZnS	170.8	5000	5000	SIBs	12

DT-C	246<br>153	100<br>10000	1000<br>5000	SIBs	13
$SnO_2/BaTiO_3$ heterostructures	183.4	10000	5000	SIBs	14

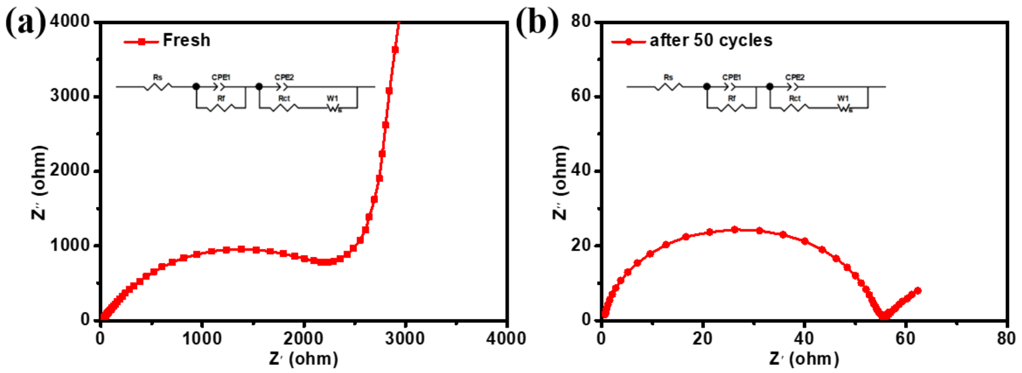

Fig. S21. Nyquist plots of $Sn_3(PO_4)_2$@PC-48 before and after 50 cycles.

Table S3 Fitting results of the EIS spectra in Fig. S14.

Electrode	R_s/Ω	R_f/Ω	R_{ct}/Ω
$Sn_3(PO_4)_2$@PC-48 before cycles	7.6	2989	1398
$Sn_3(PO_4)_2$@PC-48 after 50 cycles	9.2	48.7	57.6

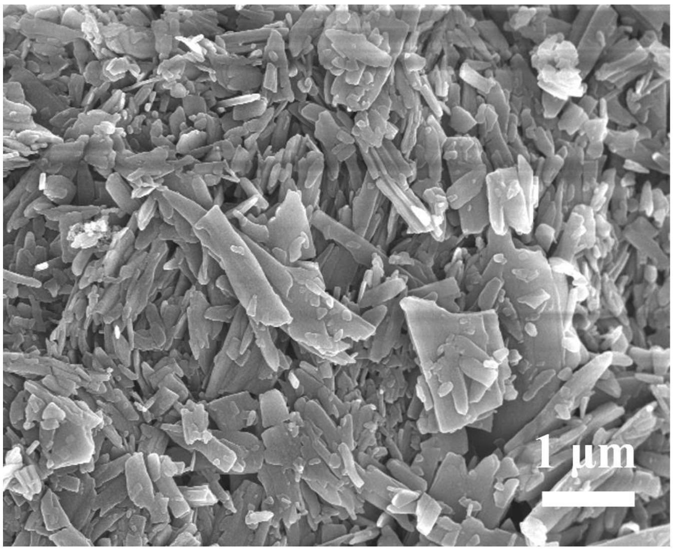

Fig. S22. Typical SEM images of $Sn_3(PO_4)_2$@PC-200 after 300 cycles.

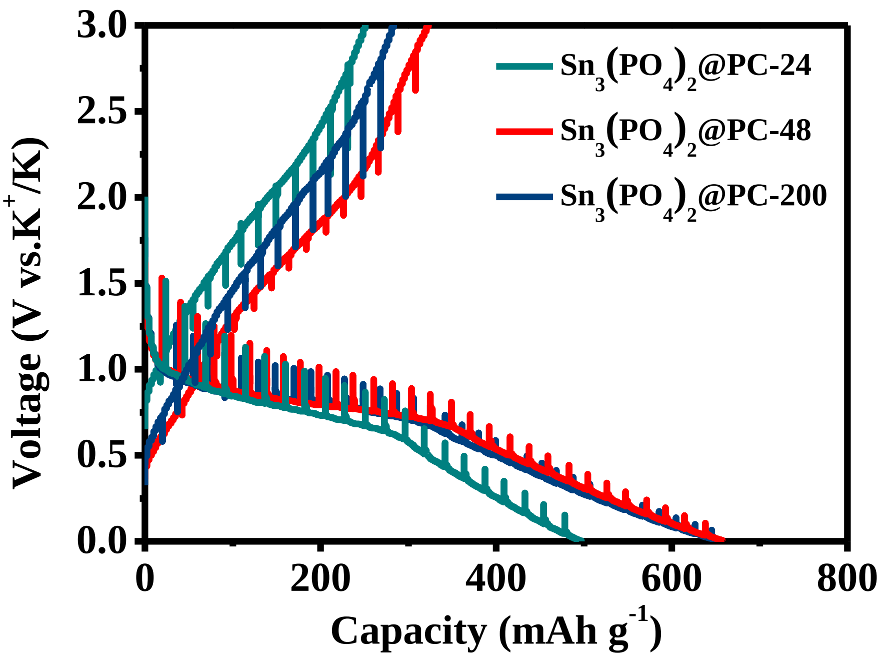

Fig. S23. Electrochemical kinetic analyses of $Sn_3(PO_4)_2$@PC by GITT.

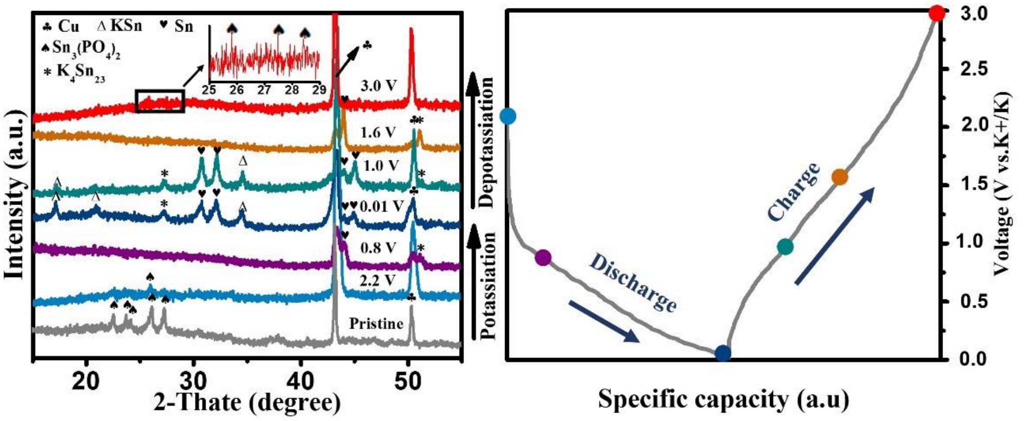

Fig. S24. Ex situ XRD patterns of the $Sn_3(PO_4)_2$@PC-48 electrode at different charge/ discharge states of PIBs.

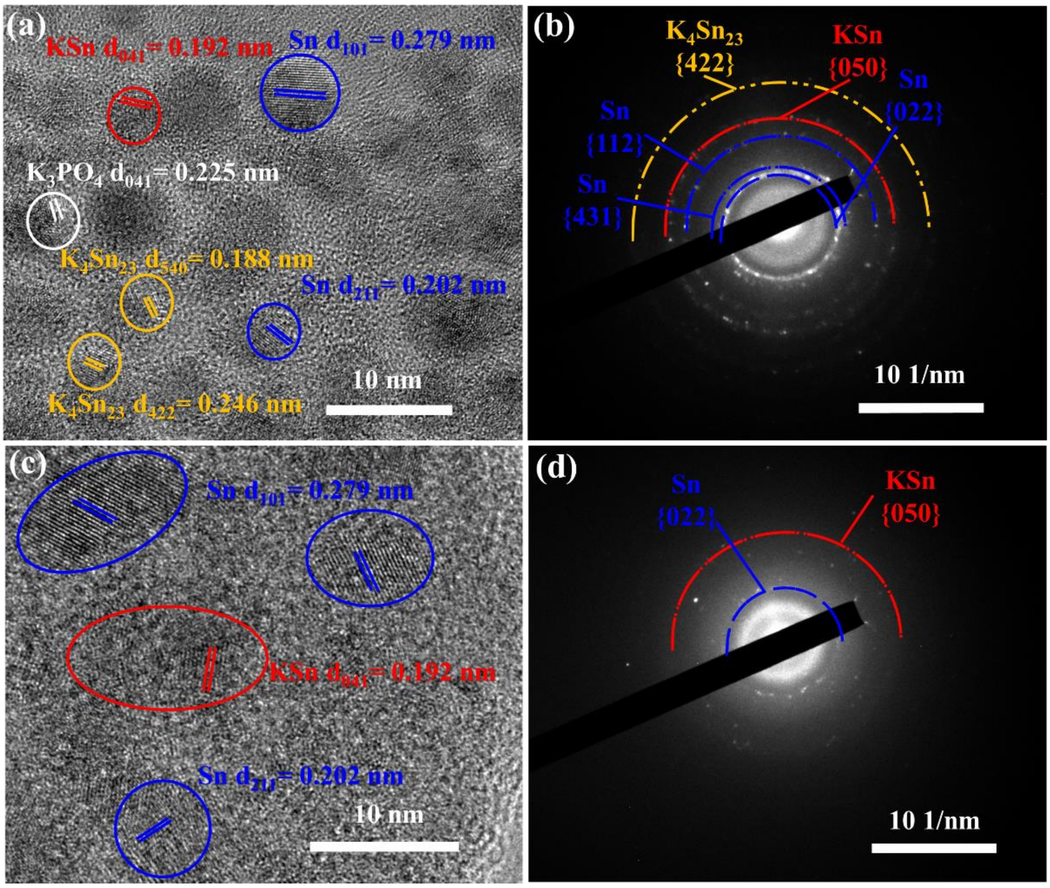

Fig. S25. a) TEM image and b) SAED pattern of the fully discharged $Sn_3(PO_4)_2$@PC-48 electrode. c) TEM image and d) SAED pattern of the fully charged $Sn_3(PO_4)_2$@PC-48 electrode.

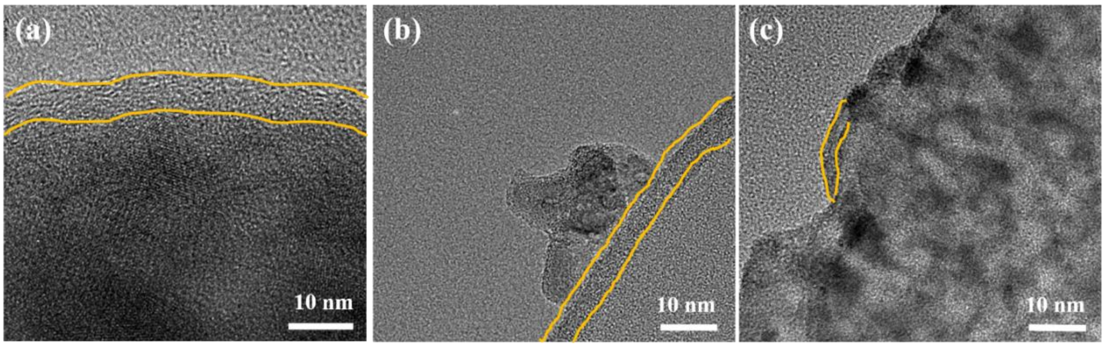

Fig. S26. The TEM images of (a) $Sn_3(PO_4)_2$@PC-24, (b) $Sn_3(PO_4)_2$@PC-24 and (c) $Sn_3(PO_4)_2$@PC-200 after 300 cycles.

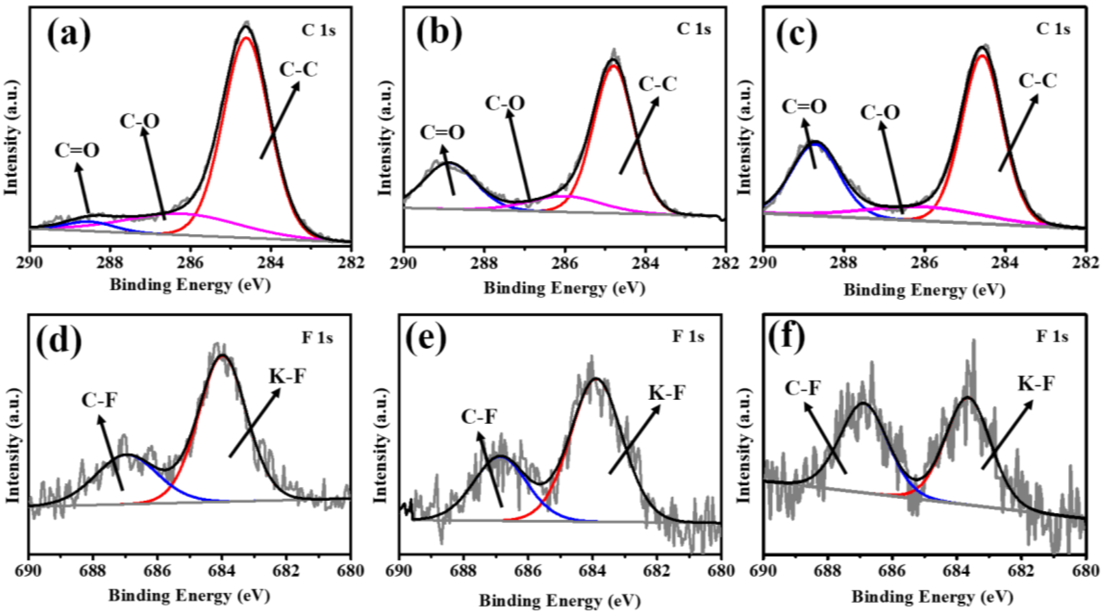

Fig. S27. High-resolution C 1s XPS spectra of (a) $Sn_3(PO_4)_2$@PC-48, (b) $Sn_3(PO_4)_2$@PC-24 and (c) $Sn_3(PO_4)_2$@PC-200 after 300 cycles. High-resolution F 1s XPS spectra of (c) $Sn_3(PO_4)_2$@PC-48, (d) $Sn_3(PO_4)_2$@PC-24 and (e) $Sn_3(PO_4)_2$@PC-200 after 300 cycles.

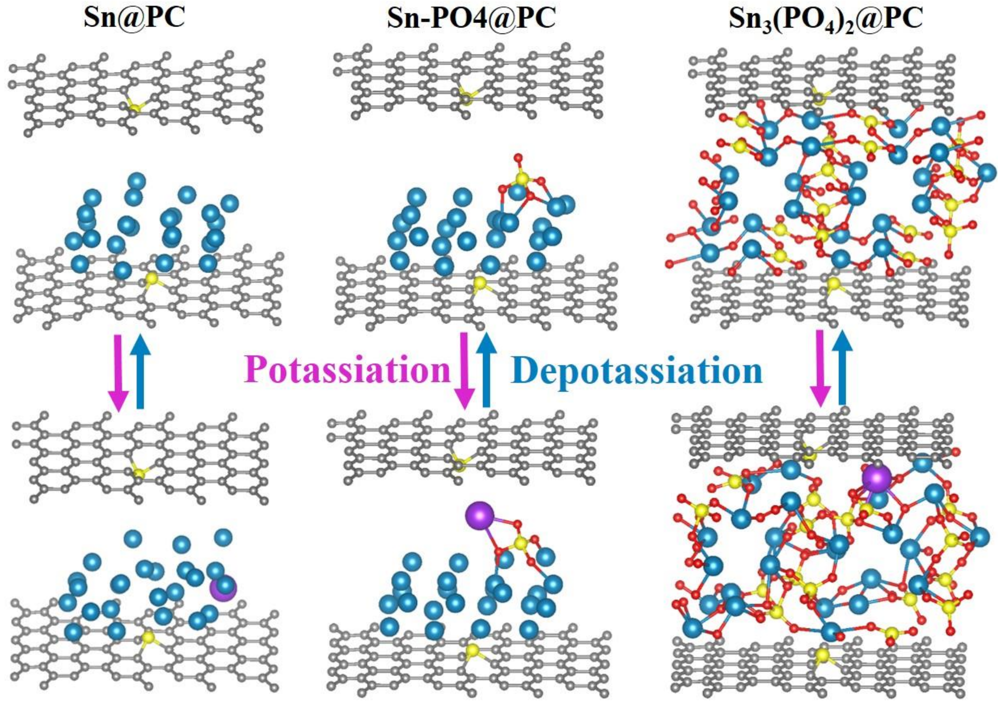

Fig. S28. Structure models of the samples at potassiation and depotassiation states.

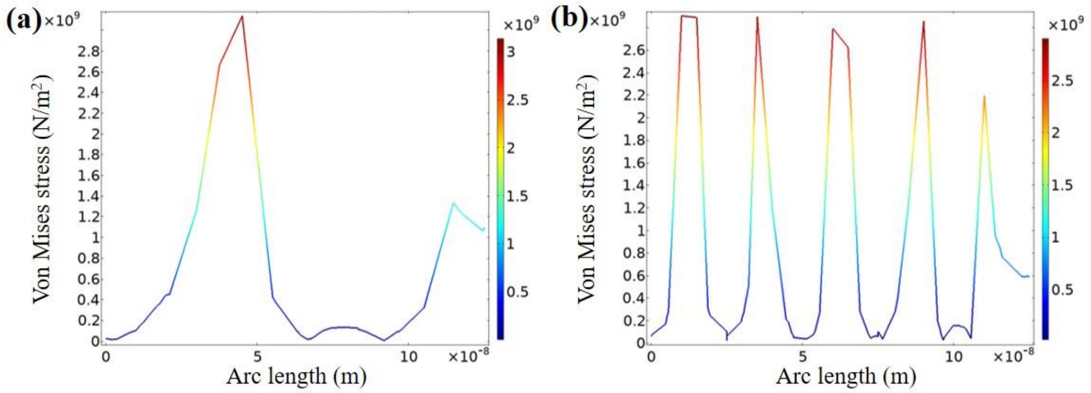

Fig. S29. The linear distributions of the Von Mises stress on $Sn_3(PO_4)_2$@PC-200 and $Sn_3(PO_4)_2$@PC-48 structural units.

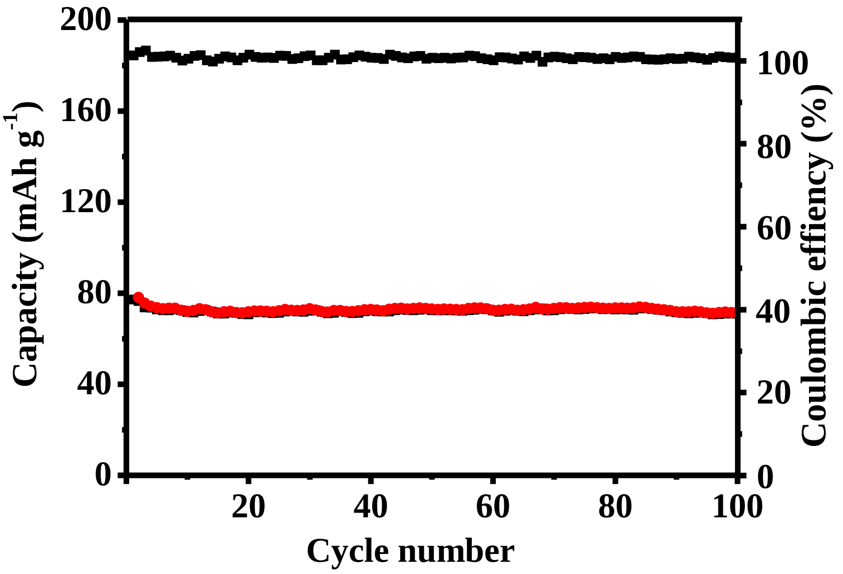

Fig. S30. The long-term stability of $Sn_3(PO_4)_2$@PC-48//PB potassium-ion full cell under 0.5 A g^{-1} ranging from 0.8 to 3.2 V.

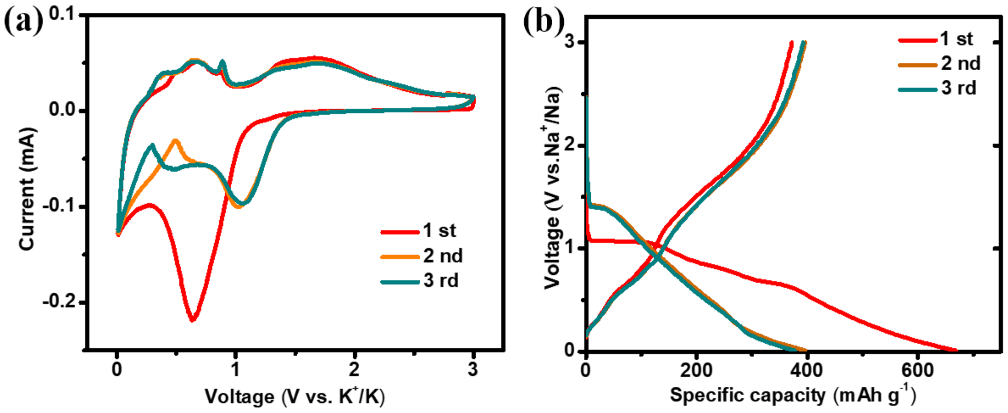

Fig. S31. Electrochemical performances for SIBs. a) CV curves of the $Sn_3(PO_4)_2$@PC-48 electrode at a scan rate of 0.1 mV s^{-1} for the first three cycles. b) Galvanostatic discharge and charge profiles for the first three cycles of the $Sn_3(PO_4)_2$@PC-48 electrode at a current density of 0.1 A g^{-1}.

Note: Fig. S31a displays the CV curves at the scan rate of 0.1 mV s^{-1} and charge/discharge profiles at 100 mA g^{-1} of $Sn_3(PO_4)_2$@PC-48 between 3.0 and 0.01 V (vs Na^+/ Na). In the first cathodic process, the cathodic peak at 0.63 V is related to the

reduction of $Sn_3(PO_4)_2$ to metallic Sn, the decomposition of electrolytes, the alloying of Sn-Na, as well as the formation of solid electrolyte interphase (SEI) film. Another cathodic peak at the potential of 0.01 V can be ascribed to the further alloying reaction and the intercalation of Na^+ into carbon substance. Then, as the electrode is charged back to 3.0 V, Na ions are extracted from carbon and Na_xSn, resulting in the two peaks at 0.6 and 0.9 V, the peak at 1.8 V is likely nested in the oxidation of Sn to $Sn_3(PO_4)_2$. In the second cycle, the cathodic peak corresponding to the reduction from $Sn_3(PO_4)_2$ to metallic Sn moves to 1.0 V, indicating the reduced polarization for this reaction. This movement also renders the alloying reaction of Sn-Na to appear at 0.6 V and 0.85 V. Meanwhile, the cathodic peak close to the discharging end is kept, confirming the good reversibility of the intercalation reaction in carbon. The CV curves and charge/ discharge profiles became almost identical from the second cycle, suggesting excellent reversibility of the $Sn_3(PO_4)_2$@PC-48 electrode. The galvanostatic charge/ discharge profiles of the $Sn_3(PO_4)_2$@PC-48 electrode at a current density of 0.1 A g^{-1} are presented in **Fig. S31b**.

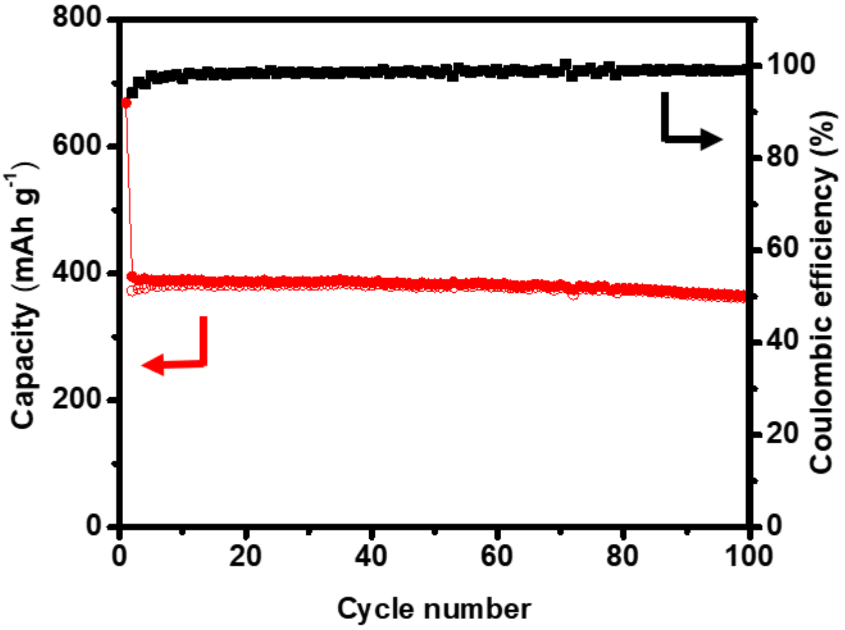

Fig. S32. Cycle performance of the $Sn_3(PO_4)_2$@PC-48 electrodes for Na ion storage at 0.1 A g^{-1}.

Note: The cycle performances of the $Sn_3(PO_4)_2$@PC-48 electrode for Na ion storage at 0.1 A g^{-1} is shown in **Fig. S32**. The initial discharge and charge capacities of the $Sn_3(PO_4)_2$@PC-48 electrode were measured to be 669.2 and 372.8 mAh g^{-1}, respectively, exhibiting an initial CE of 55.7%. In the following cycles, a reversible capacity of 380 Ah g^{-1} can be achieved.

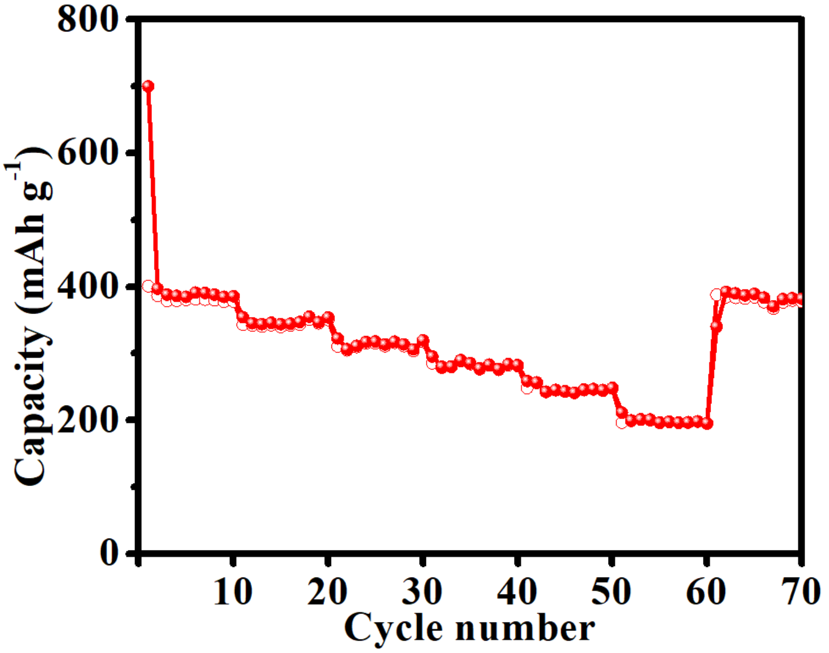

Fig. S33. Rate performance of $Sn_3(PO_4)_2$@PC-48 for SIBs.

Note: The rate capabilities of the $Sn_3(PO_4)_2$@PC-48 electrodes were investigated by cycling the electrodes at different current densities in sequence (**Fig. S33**). The $Sn_3(PO_4)_2$@PC-48 electrode exhibits outstanding reversible capacities of 386, 343, 310, 285, 248 and 201 mAh g^{-1} at 0.1, 0.2, 0.5, 1, 2 and 5 A g^{-1}, respectively. Furthermore, when the current density is reset to 0.1 A g^{-1}, the capacity can fully recover.

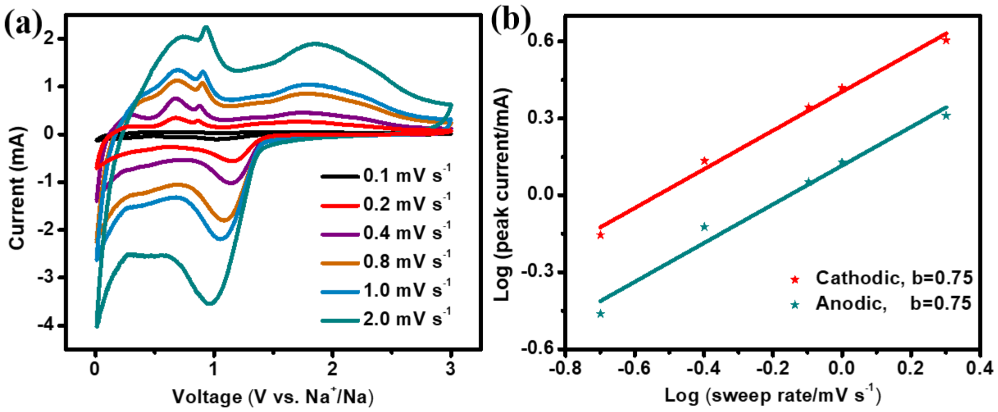

Fig. S34. Electrochemical performances for SIBs. a) CV curves of the $Sn_3(PO_4)_2$@PC-48 electrode at various scan rate from 0.1 to 2 mV s^{-1}. b) Plots of log (scan rate) versus log (peak current).

Note: To further investigate the kinetics of sodium insertion/ extraction into/ from the electrode, a series of CV curves with various scan rates of 0.1, 0.2, 0.4, 0.8, 1.0, and 2.0 mV s^{-1} were recorded to investigate the Na^+ diffusion in $Sn_3(PO_4)_2$@PC-48 electrodes (**Fig. S34a**). According to the fitted results (**Fig. S34b**), the value of *b* is calculated to be 0.75 for $Sn_3(PO_4)_2$, indicating dominated surface capacitive storage in the $Sn_3(PO_4)_2$@PC-48 electrode. As illustrated in **Fig. S35a**, the capacitive process contributes 82.9% of the total capacity at a scan rate of 1.0 mV s^{-1}. These data gradually decrease to 71.3% as the scan rate slows to 0.2 mV s^{-1} and increase to 85.3% at the scan rate of 2.0 mV s^{-1} (**Fig. S35b**).

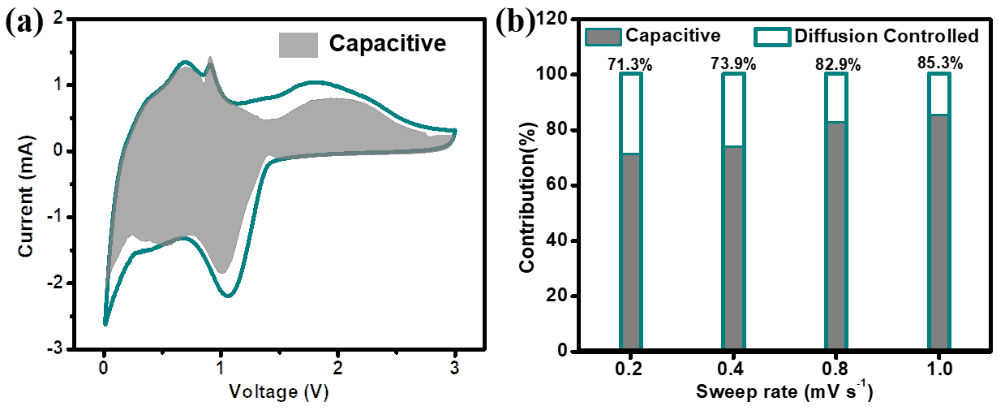

Fig. S35. a) Capacitive contribution in CV curves (shaded region) of $Sn_3(PO_4)_2$@PC-48, b) Contribution ratio of capacitive capacity in $Sn_3(PO_4)_2$@PC-48 at different sweep rates in SIBs.

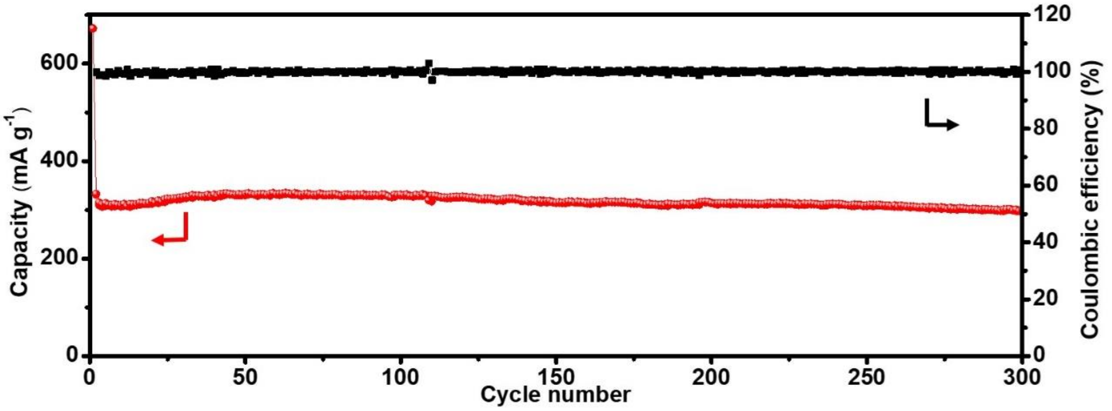

Fig. S36. Long-term cycling performance of $Sn_3(PO_4)_2$@PC-48 at a current density of 0.5 A g^{-1}.

Note: The long-term cycling performance of $Sn_3(PO_4)_2$@PC-48 at 0.5 A g^{-1} was further investigated, as shown in **Fig. S36**. The $Sn_3(PO_4)_2$@PC-48 anodes delivered a capacity of 309.9 mAh g^{-1} at the second cycle (the initial discharge capacity is neglected because of the formation of an SEI during the first discharge process) and remained 99% (305.8 mAh g^{-1}) after 300 cycles at 0.5 A g^{-1}.

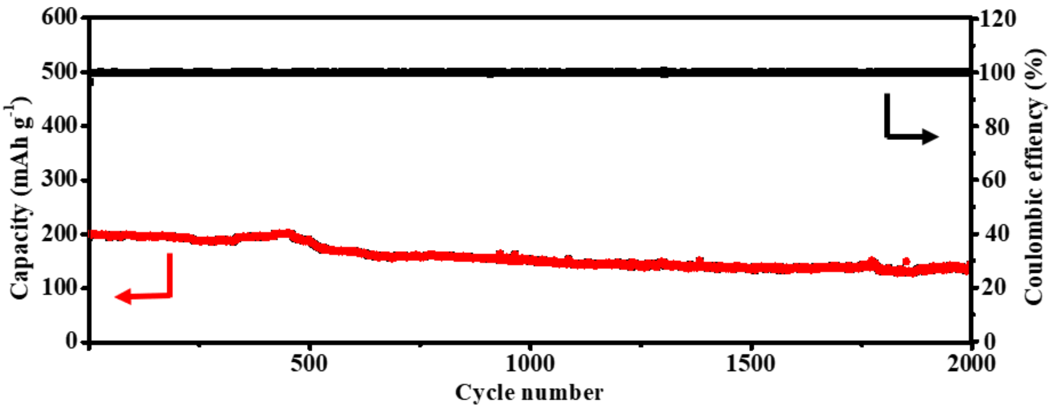

Fig. S37. Cycle performance of the $Sn_3(PO_4)_2$@PC-24 electrodes for Na ion storage at 5 A g^{-1}.

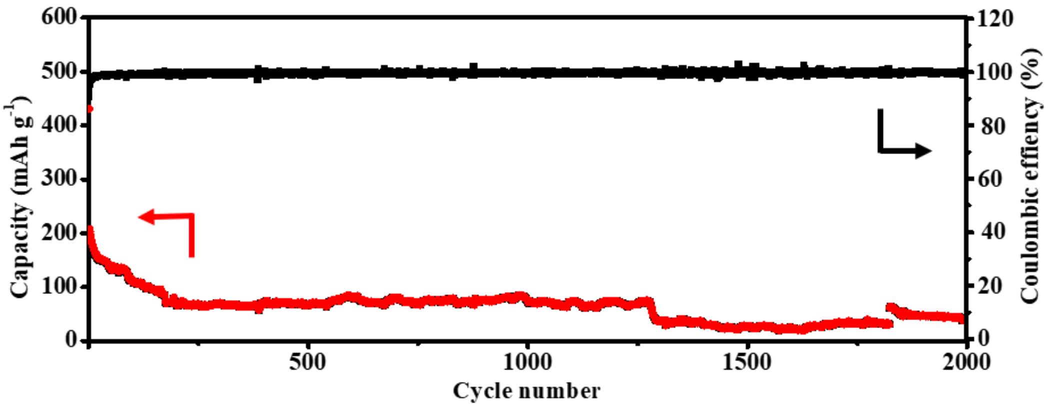

Fig. S38. Cycle performance of the $Sn_3(PO_4)_2$@PC-200 electrodes for Na ion storage at 5 A g^{-1}.

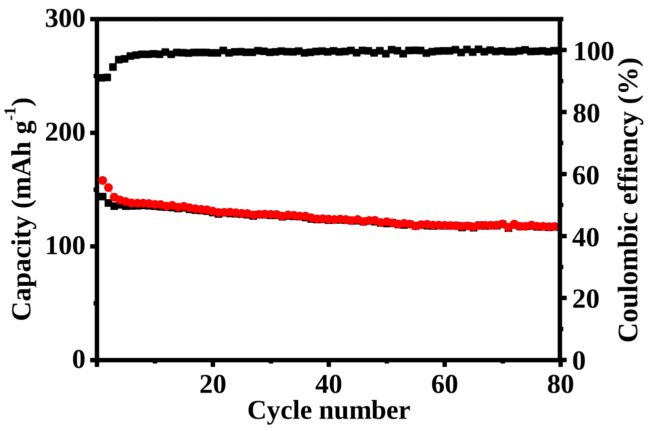

Fig. S39 The long-term stability of $Sn_3(PO_4)_2$@PC-48//PB sodium-ion full under 0.5 A g^{-1} ranging from 0.8 to 3.2 V.

References

1. N. Stock, N. Guillou, T. Bein and G. Férey, *Solid State Sciences*, 2003, **5**, 629-634.
2. N. Cheng, W. Zhou, J. Liu, Z. Liu and B. Lu, *Nano-Micro Letter*, 2022, **14**, 146.
3. Q. Zhang, X. Cheng, C. Wang, A. M. Rao and B. Lu, *Energy & Environmental Science*, 2021, **14**, 965-974.
4. H. Qiu, L. Zhao, M. Asif, X. Huang, T. Tang, W. Li, T. Zhang, T. Shen and Y. Hou, *Energy & Environmental Science*, 2020, **13**, 571-578.
5. C. Xu, J. Mu, T. Zhou, S. Tian, P. Gao, G. Yin, J. Zhou and F. Li, *Advanced Functional Materials*, 2022, **32**, 2206501.
6. X. Xu, Y. Zhang, H. Sun, J. Zhou, Z. Liu, Z. Qiu, D. Wang, C. Yang, Q. Zeng, Z. Peng and S. Guo, *Advanced Materials*, 2021, **33**, 2100272.
7. H. Shan, J. Qin, Y. Ding, H. M. K. Sari, X. Song, W. Liu, Y. Hao, J. Wang, C. Xie, J. Zhang and X. Li, *Advanced Materials*, 2021, **33**, 2102471.
8. Y. Qian, S. Jiang, Y. Li, Z. Yi, J. Zhou, J. Tian, N. Lin and Y. Qian, *Energy Storage Materials*, 2020, **29**, 341-349.
9. J. L. Xia, D. Yan, L. P. Guo, X. L. Dong, W. C. Li and A. H. Lu, *Advanced Materials*, 2020, **32**, 2000447.
10. Z. Hu, Q. Liu, W. Lai, Q. Gu, L. Li, M. Chen, W. Wang, S. L. Chou, Y. Liu and S. X. Dou, *Advanced Energy Materials*, 2020, **10**, 1903542.
11. B. Sun, S. Lou, W. Zheng, Z. Qian, C. Cui, P. Zuo, C. Du, J. Xie, J. Wang and G. Yin, *Nano Energy*, 2020, **78**, 105366.
12. Y. Li, J. Qian, M. Zhang, S. Wang, Z. Wang, M. Li, Y. Bai, Q. An, H. Xu, F. Wu, L. Mai and C. Wu, *Advanced Materials*, 2020, **32**, 2005802.
13. R. Guo, C. Lv, W. Xu, J. Sun, Y. Zhu, X. Yang, J. Li, J. Sun, L. Zhang and D. Yang, *Advanced Energy Materials*, 2020, **10**, 1903652.
14. R. Li, G. Zhang, Y. Wang, Z. Lin, C. He, Y. Li, X. Ren, P. Zhang and H. Mi, *Nano Energy*, 2021, **90**, 106591.
